# Is Living in a U.S. Coastal City Good for One’s Health?

**DOI:** 10.3390/ijerph18168399

**Published:** 2021-08-09

**Authors:** Paul A. Sandifer, Alexander S. Braud, Landon C. Knapp, Judith Taylor

**Affiliations:** 1 Center for Coastal Environmental and Human Health, College of Charleston, Charleston, SC 29424, USA; braudas@cofc.edu (A.S.B.); knapplc@cofc.edu (L.C.K.); taylorj4@g.cofc.edu (J.T.); 2 San Francisco Estuary Institute, Richmond, CA 94804, USA

**Keywords:** coasts, health, urban areas, Gulf of Mexico, blue space, BRFSS, healthy days

## Abstract

Background: Evidence suggests that living close to “blue spaces” (water features), particularly coastlines, has salutary effects on human health. Methods: We analyzed five years of annual, self-reported general health and unhealthy days data from the Behavioral Risk Factor Surveillance System of the U.S. Centers for Disease Control and Prevention for 165 urban areas across the contiguous U.S. We compared health self-reports for people living in coastal vs. non-coastal urban areas and for residents of the disaster-prone Gulf of Mexico region vs. other locations. Coastal urban areas were defined as those having ≥50% of their population living within 20 km of a coast. Results: We found no overall health advantage of residing in a coastal urban location when all urban areas were considered. However, residents from non-Gulf of Mexico coastal urban areas reported modestly better health than residents from non-coastal areas. In contrast, self-reported health of Gulf coastal urban residents was significantly poorer than that of residents from other urban areas. Conclusions: The frequency of disasters and history of health and socioeconomic disparities in the Gulf region may be responsible, at least in part, for the apparent lack of health promoting effects of coastal location there.

## 1. Introduction

A substantial amount of literature provides evidence that living in proximity to “blue spaces,” that is, natural and man-made water features, particularly coastlines, has salutary effects on human health [1,2,3,4,5,6,7,8]. According to these studies, positive effects are strongest for mental health, especially restoration and reduction in stress, anxiety, and depression. Physical health effects are less apparent, with most related to walking and other forms of outdoor exercise [8]. In general, it appears that the closer one is to the coast, either for residence or visitation, the stronger the positive health effects [7,9]. These effects may be more pronounced and important for economically and otherwise disadvantaged people [10] and those who have actual views of the coast [8].

While potential health-promoting effects of coastal living are now recognized (bluehealth2020.eu (accessed on 3 August 2021)), the numbers and intensity of environmental disasters including those affecting coastal areas are apparently increasing [11]. These, along with related and separate effects of climate change, have raised grave concerns about the long-term survivability of some coastal communities [12,13,14,15]. The U.S. Gulf of Mexico (Gulf) coast has been particularly affected by recurrent hurricanes and tropical storms, including six that made landfall in 2020 alone. Additionally, the region has been imperiled by the catastrophic Deepwater Horizon (DWH) oil spill of 2010, numerous harmful algal blooms, and other disasters, to such a degree that it was deemed critical to design a disaster-focused human health observing system for the region [16,17]. Thus, while some coastal environments may offer important health benefits, it is also apparent that others may, at times, present numerous threats to human health and well-being [18,19,20]. Some of the latter are being investigated via the Centers for Oceans and Human Health in the U.S. (https://www.niehs.nih.gov/research/supported/centers/oceans/index.cfm last accessed on 3 August 2021). 

The cross-sectional Behavioral Risk Factor Surveillance Survey (BRFSS) (https://www.cdc.gov/brfss/index.html last accessed on 3 August 2021) is a continuous, state-based, random telephone survey of community dwelling U.S. adults aged 18 and older that is implemented annually by state health departments in the U.S. under the auspices of the Centers for Disease Control and Prevention (CDC). While the survey contains mandatory core sections, states have flexibility to add questions and extend data collection to include additional participants to address health issues of particular concern to one or more states. An example is the extended BRFSS survey implemented as the Gulf States Population Survey (GSPS) following the DWH event [21,22,23]. BRFSS data are commonly used in health studies at state and national levels, e.g., [24,25,26,27,28]. Health-related quality of life (HRQOL or Healthy Days) questionnaires are one of the survey’s strongest features, allowing comparisons within large populations and across time and geography [29,30]. The proportion or number of mentally or physically unhealthy days in particular have been widely used to compare health of populations [30,31,32,33,34] (http://www.cdc.gov/hrqol last accessed on 3 August 2021). Healthy Days data are the focus of the present study. Unfortunately, the sampling density of national surveys such as the BRFSS is usually insufficient to detect effects of individual disasters at the population level. Thus, we did not attempt to evaluate effects of disasters per se, but instead explore whether the Gulf of Mexico region, with its long history of environmental issues, might reflect different health responses than other areas of the country.

In their comprehensive review, White et al. [8] found substantial evidence supporting a “blue space”–health connection but did not draw firm conclusions due to several study limitations, including lack of discovery of studies with null or negative effects and of consideration of health risks that may be associated with coastal areas, such as repeated exposure to environmental disasters as experienced by coastal residents of the Gulf of Mexico region. In addition, they did not explore specifically whether living in coastal urban areas per se might or might not affect health, although they noted that throughout history, human towns and cities often have been located close to coasts or inland water bodies. The present study was designed to address these gaps, at least in part, through exploration of the following research questions: (1) Do people who live in U.S. coastal urban areas report better health than those who live in non-coastal urban areas? (2) Do residents of urban areas on the disaster-prone U.S. Gulf of Mexico coast report poorer health than their counterparts who live in coastal or non-coastal urban areas elsewhere in the U.S.?

## 2. Materials and Methods

### 2.1. BRFSS MMMSA Data and Selection and Categorization of MMSAs 

We used data derived from the BRFSS Selected Metropolitan/Micropolitan Statistical Area Risk Trends (SMART) for the contiguous U.S. as these data are readily available for several years, reflect residential circumstances for a substantial portion of the U.S. population (~81% of US residents live in urban areas) [35], and have been used successfully in other health-related studies [36,37,38,39,40,41]. BRFSS SMART data are compiled by the CDC for geographic subdivisions smaller than states and termed MMSAs (metropolitan statistical areas, micropolitan statistical areas, and metropolitan divisions) by the U.S. Census Bureau (https://www2.census.gov/geo/tiger/TGRGDB20/tlgdb_2020_a_us_substategeo.gdb.zip last accessed on 3 August 2021). Metropolitan statistical areas represent groups of counties including at least one urban area with >50,000 residents, micropolitan statistical areas are groups of counties with at least one urban area having a population of >10,000 but <50,000 residents, and metropolitan divisions are groups of counties within a metropolitan statistical area containing >2.5 million residents. The threshold for inclusion of a specific MMSA in BRFSS SMART data is at least 500 survey respondents to the annual BRFSS survey from that area. All data utilized in this study are publicly available from the CDC (https://www.cdc.gov/brfss/smart/Smart_data.htm last accessed on 3 August 2021). 

We are not aware of any universally accepted definition of what constitutes a “coastal city.” In the US, coastal areas are typically described in terms of counties that border an ocean, the Gulf of Mexico, or the Great Lakes or encompass areas at high risk for tidal and/or storm surge flooding as identified by the Federal Emergency Management Agency (termed shoreline counties). An additional tier of U.S. coastal counties, termed watershed counties, are those that are immediately landward of the shoreline counties [42]. Alternatively, the EU considers anyone living <50 km from a coast to be coastal residents and those residing >50 km to be inland [7]. Because many of the MMSAs have a large geographic footprint that may extend over all or parts of multiple counties (e.g., Atlanta, GA, USA), and/or may extend well inland from the actual coast (e.g., Houston, TX, USA), we refined our definition of coastal to more clearly identify those areas with substantial human populations in relatively close proximity to coastal shorelines. Unfortunately, the BRFSS data sets did not allow determination of respondents’ actual distances from a coast. Previous studies of coastal location and health have found positive results only for relatively close coastal proximity, e.g., <20 km. Consequently, after considering several options, we restricted our definition of coastal MMSAs to those with >50% of their population lying within 20 km of a coast. 

**Figure 1:**
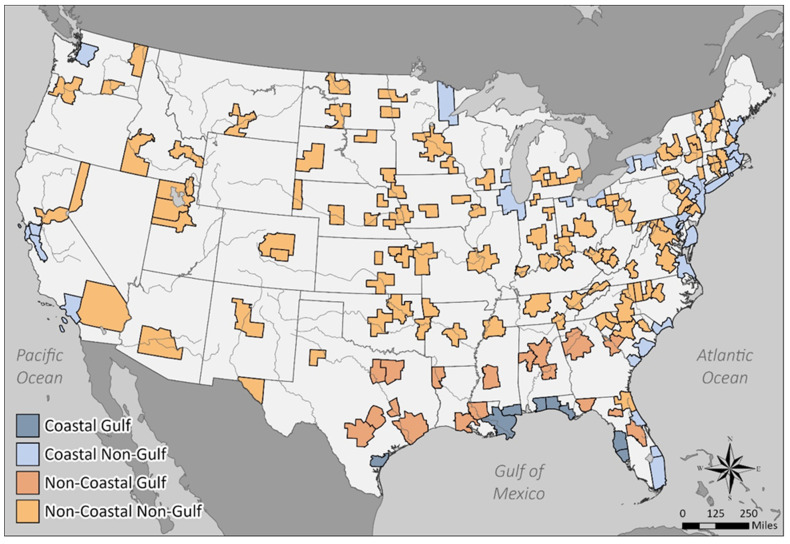
Map of the continental United States showing the location of all 165 MMSAs included in the study.

This coastal categorization resulted in classification of a number of cities as non-coastal MMSAs that otherwise might be considered “coastal” in terms of general location (Figure 1, Table S1) because of the substantial distance of many of their residents from the actual coast. An assumption of this study is that the majority of respondents in those MMSAs would not experience the putative health influence of living near the coast. MMSA population distribution and shoreline boundary were analyzed using the U.S. Environmental Protection Agency EnviroAtlas Dasymetric Allocation of Population [43,44] and the Global Self-consistent, Hierarchical, High-resolution Geography Database [45] (data version 2.3.7 released 15 June 2017), respectively. 

BRFSS SMART data were analyzed for the five-year period 2013–2017, with a total of 165 MMSAs containing sufficient data for analysis during that timeframe (Table S1, Figure 1). There were some differences in inclusion of MMSAs from year to year (Table S1). The mean number of MMSAs included was 134 per year (range, 127–141) and a total of 104 were included in all years. According to the CDC, variations in MMSA inclusion may reflect differences in weighting factors from year to year or failure of some to meet the minimum 500 interview criterion in a given year. Thirty-seven MMSAs met our coastal criteria discussed above and 128 were categorized as non-coastal. Of the 37 coastal MMSAs, 8 were in the Gulf of Mexico region and 29 were non-Gulf coastal. Similarly, of the 128 non-coastal MMSAs, 18 were considered as part of the Gulf region and 110 non-Gulf non-coastal. The Gulf region was characterized as MMSAs within 400 km of the Gulf of Mexico coastline and not bordering the Atlantic Ocean.

While the BRFSS survey encompasses a broad range of health questions, this study focused on self-reported health metrics based on responses to the four BRFSS Healthy Days questions. The Healthy Days questions are as follows:

1. Would you say that in general your health is excellent, very good, good, fair, or poor?
2. Now thinking about your physical health, which includes physical illness and injury, for how many days during the past 30 days was your physical health not good?
3. Now thinking about your mental health, which includes stress, depression, and problems with emotions, for how many days during the past 30 days was your mental health not good?
4. During the past 30 days, for about how many days did poor physical or mental health keep you from doing your usual activities, such as self-care, work, or recreation?

Types and abbreviations for responses to these questions are listed in Table 1. These abbreviations are used throughout the remainder of the text and tables. 

Although all of these metrics have been widely used (http://www.cdc.gov/hrqol last accessed on 3 August 2021), the majority of responses tend to fall into the E, VG, G or 0-days categories. The F, P and ≥14-day ratings are generally diagnostic of significant mental or physical health issues [29,30,46]. The 1–13 days category appears to have been less used in prior analyses [29,47], and, although the data were examined, this category was not considered in our interpretations. We followed other researchers [29,34,48] by further grouping the results for GH into E/VG/G and F/P, which are complementary, as well as emphasizing the 0- and ≥14-days categories of unhealthy days.

Here, we compared GH and unhealthy days for all coastal MMSAs versus all non-coastal, Gulf coastal versus Gulf non-coastal MMSAs, Gulf coastal MMSAs versus all coastal MMSAS, and non-Gulf coastal MMSAs versus all non-Gulf non-coastal MMSAs. 

### 2.2. Analytical Approaches and Statistics

A major consideration for understanding and evaluating BRFSS data is recognizing that the system is not a simple random sample of the population. Instead, BRFSS consists of respondents from a complex survey using a multistage design to gather a representative sample from each state’s noninstitutionalized civilian resident population. Because of the complex design, the direct application of standard statistical methods for variance estimation and hypothesis testing may yield misleading results (https://www.cdc.gov/brfss/annual_data/2019/pdf/compare-2019-508.pdf last accessed on 3 August 2021). The BRFSS sampling design includes stratification, clustering, and weighting, each of which must be accounted for in statistical analyses. Through the system’s weighting and raking approaches, each respondent is given a weight to represent how common characteristics of an individual are within the population. Respondent characteristics taken into consideration include age, sex, race/ethnicity, region, telephone ownership, education level, marital status, and home ownership. Additional raking is performed in production of the SMART data to create the MMSA weights used in our analysis. Due to the complex sampling design, special consideration and software are required to appropriately infer statistical results from the data.

For analyses, we used the Complex Samples Add-On to the IBM SPSS software (IBM Corporation, Armonk, NY, USA) package. This allowed for the complex sampling plan and respondent weighting to be appropriately defined and considered. One of the advantages of the BRFSS SMART data is that the data, as aggregated and weighted by the CDC, allow statistical comparisons among the MMSAs. Our efforts strictly involved exploring frequency and descriptive statistics with the coastal/non-coastal or Gulf/non-Gulf classification as the defined subpopulations. Following the approach recommended by the CDC, we considered differences in means significant at *p* < 0.05 when the confidence intervals (CIs) did not overlap and those with overlapping CIs were considered non-significant (https://www.cdc.gov/brfss/smart/smart_faq.htm accessed on 3 August 2021, see section on comparing prevalence). There were several instances where the CI’s met but did not overlap; we conservatively considered those the same as overlapping, that is, non-significant. While more advanced statistical analyses are possible with the Complex Samples Add-On, the skewed distribution of healthy days-related questions can cause misleading results [49]. We chose the conservative CI difference approach as our primary focus was restricted to the question of self-reported health status in relation to coastal or non-coastal residency. 

To assess possible underlying influences on health-related questions, similar comparative statistics were performed on demographic characteristics, including gender, race/ethnicity, age, education, and income. However, due to the potential misleading results linear regression can have on count data like the unhealthy days responses [49] and considering the limitations of the SPSS Complex Samples Add-On, analysis of covariates was not pursued. 

Limitations: While the BRFSS SMART data sets were chosen because of the national coverage, multiple years of available data, and the large size of the databases, they do not comprise all urban areas in the U.S. and not all of the MMSAs are included in all years. The absence of established definitions of what constitutes coastal and non-coastal urban areas required that we develop our own, which may have affected study results. However, we believe any effects would have been conservative, that is, reducing the possibility of finding location-based differences. Additionally, the comparatively few MMSAs in the Gulf, plus the relatively high level of potential impact of a few large MMSAs, such as the Tampa-St. Petersburg-Clearwater (Tampa) MMSA in the Gulf, may have the potential to skew findings. To evaluate the latter possibility, we analyzed the Gulf coast MMSA data without Tampa vs. with Tampa since Tampa had the largest weight within any MMSA grouping in the study. Results indicated that inclusion or exclusion of Tampa had no significant effect on any health measure for any of the years studied. Based on this finding, we assumed that any other somewhat highly weighted areas would also have little to no effect on the outcomes. Another limitation of the BRFSS data is the lack of a way to estimate actual exposure to blue spaces other than residence within an urban area that is located along or relatively near a coastline. Thus, one cannot assess the actual proximity of participants to a coast or their potential to access blue spaces either for visits or incidentally during commutes or other local travel or to assess likely pathways by which health may be affected by coastal exposure. Finally, because the data are limited to self-rated responses from cross-sectional studies, they can only be considered as indicative.

## 3. Results

**Figure 2:**
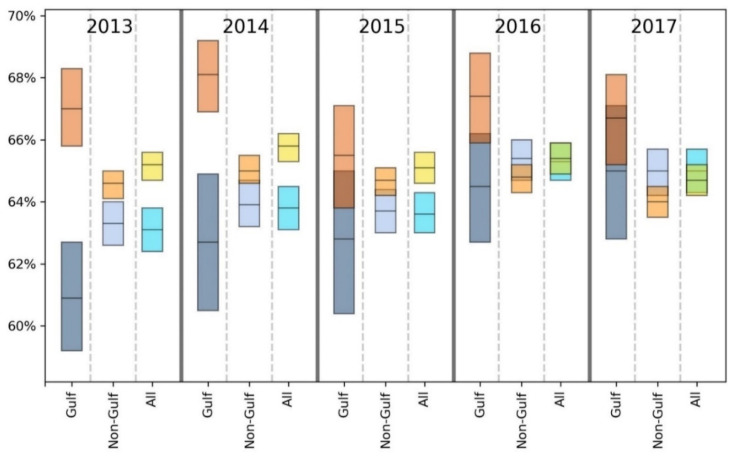
Comparison of mean (black line across bar) and 95% confidence intervals for percentages of Gulf and non-Gulf MMSA respondents reporting zero physically unhealthy days out of the last 30. Colors denote the following: dark blue = coastal Gulf, light blue = coastal non-Gulf, dark orange = non-coastal Gulf, light orange = non-coastal non-Gulf, bright blue = all coastal, yellow = all non-coastal.

**Figure 3:**
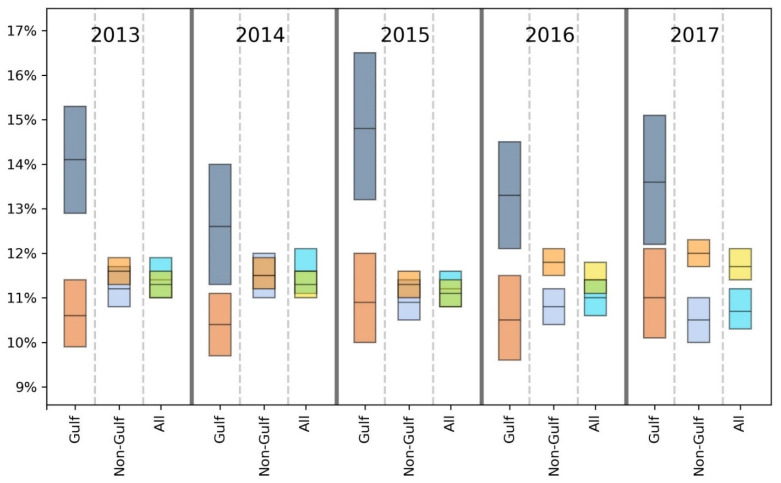
Comparison of mean (black line across bar) and 95% confidence intervals for percentages of Gulf and non-Gulf MMSA respondents reporting ≥14 physically unhealthy days out of the last 30. Colors denote the following: dark blue = coastal Gulf, light blue = coastal non-Gulf, dark orange = non-coastal Gulf, light orange = non-coastal non-Gulf, bright blue = all coastal, yellow = all non-coastal.

**Figure 4:**
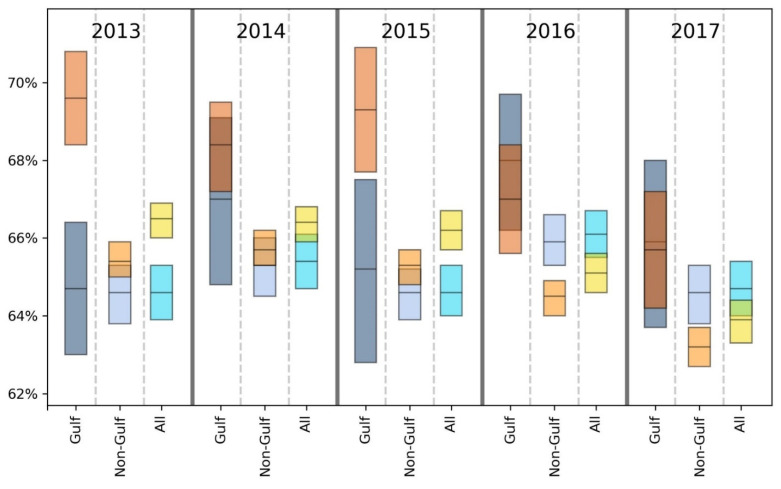
Comparison of mean (black line across bar) and 95% confidence intervals for percentages of Gulf and non-Gulf MMSA respondents reporting zero mentally unhealthy days out of the last 30. Colors denote the following: dark blue = coastal Gulf, light blue = coastal non-Gulf, dark orange = non-coastal Gulf, light orange = non-coastal non-Gulf, bright blue = all coastal, yellow = all non-coastal.

**Figure 5:**
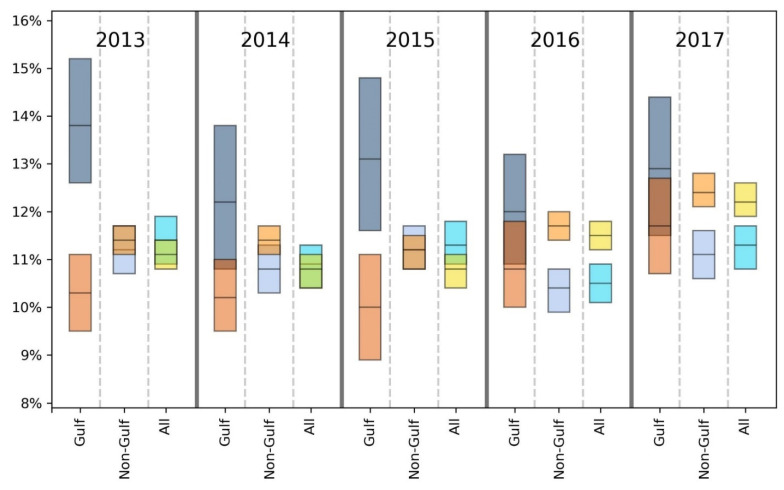
Comparison of mean (black line across bar) and 95% confidence intervals for percentages of Gulf and non-Gulf MMSA respondents reporting ≥14 mentally unhealthy days out of the last 30. Colors denote the following: dark blue = coastal Gulf, light blue = coastal non-Gulf, dark orange = non-coastal Gulf, light orange = non-coastal non-Gulf, bright blue = all coastal, yellow = all non-coastal.

**Figure 6:**
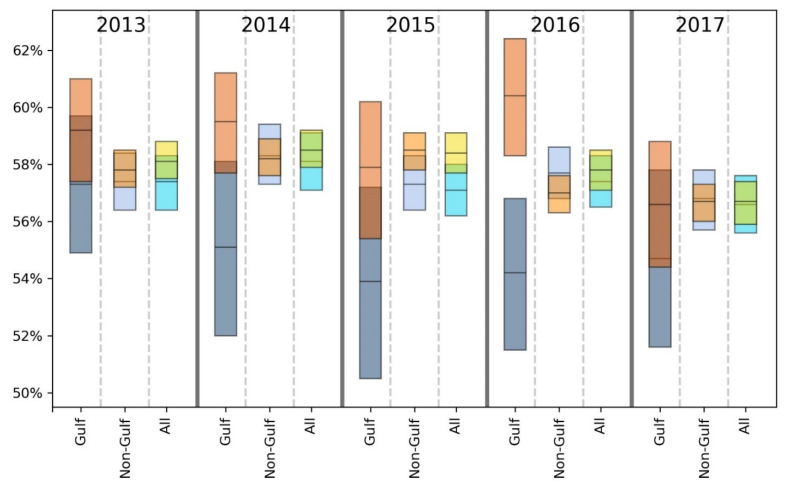
Comparison of mean (black line across bar) and 95% confidence intervals for percentages of Gulf and non-Gulf MMSA respondents reporting zero physically or mentally unhealthy days out of the last 30. Colors denote the following: dark blue = coastal Gulf, light blue = coastal non-Gulf, dark orange = non-coastal Gulf, light orange = non-coastal non-Gulf, bright blue = all coastal, yellow = all non-coastal.

**Figure 7:**
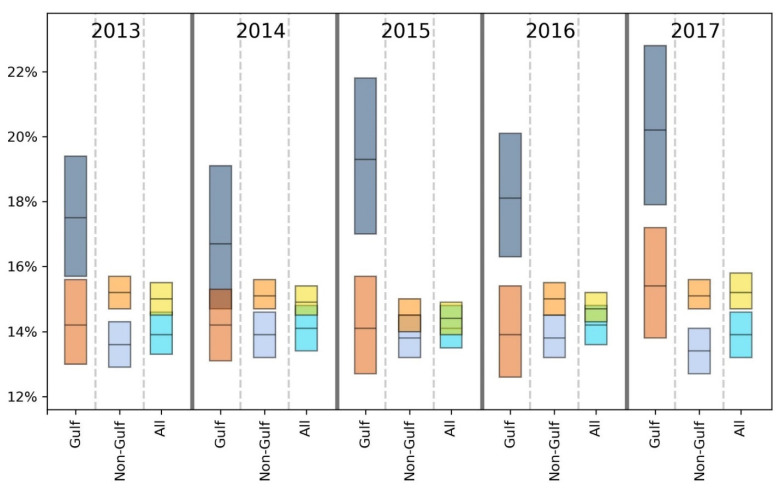
Comparison of mean (black line across bar) and 95% confidence intervals for percentages of Gulf and non-Gulf MMSA respondents reporting ≥14 physically or mentally unhealthy days out of the last 30. Colors denote the following: dark blue = coastal Gulf, light blue = coastal non-Gulf, dark orange = non-coastal Gulf, light orange = non-coastal non-Gulf, bright blue = all coastal, yellow = all non-coastal.

For brevity, we primarily discuss statistically significant findings. However, both significant and non-significant results are presented in Table 2, Table 3, Table 4, Table 5, Table 6, Table 7 and Table 8 and Figure 2, Figure 3, Figure 4, Figure 5, Figure 6 and Figure 7.

### 3.1. Comparing Health Responses for All Coastal vs. All Non-Coastal MMSAs 

When compared to non-coastal MMSAs, GH (General Health) responses from coastal MMSAs were consistently and significantly higher for the E (Excellent) health rating in 3 of 5 years (2013, 2016, 2017) and consistently lower in the VG (Very Good) rating in 4 of 5 years. The G (Good) category rarely showed any differences in comparisons throughout the study (Table 2). Interestingly, when the E/VG/G ratings were binned together, coastal MMSAs revealed significantly lower response percentages (poorer health) in 3 years (2013, 2014, 2016). Similarly, in the F (Fair) category, coastal rated significantly worse for 2013, 2014, and 2016, while the P (Poor) category results were NS for all years. The complementary E/VG/G and F/P combined ratings indicated slightly but significantly poorer reported GH for coastal residents in 3 of 5 years compared to ratings from residents of non-coastal areas. 

The percentage of coastal MMSA residents reporting 0 PUHDs (Physically Unhealthy Days) was significantly smaller than that of non-coastal respondents in 3 of 5 years (2013, 2014, 2015). In the ≥14 days category of PUHDs, which is most indicative of significant health issues [29,30], results were NS in all but 1 year (2017) when coastal responses accounted for a significantly lower proportion of unhealthy days than non-coastal. The percentage of coastal MMSA residents reporting 0 MUHDs (Mentally Unhealthy Days) was significantly lower than that of non-coastal in 2 of 5 years (2013, 2015) and significantly higher in 1 year (2016). In the ≥14-day category, coastal MMSA reports revealed significantly lower percentages of MUHDs in 2 of the 5 years (2016, 2017). For PMUHDs (Physically or Mentally Unhealthy Days), all years were NS in the 0-day category, while in the ≥14-days category, coastal had a significantly lower proportion in 1 year (2017). Considering mean numbers of unhealthy days, all results were NS for PUHDs; coastal MMSAs reported significantly fewer MUHDs in 2 years (2016, 2017); and PMUHDs were significantly lower in coastal MMSAs in 1 year (2013). The one significant difference in overall means averaged over all 5 years was for PMUHDs at 0.14 less unhealthy day for coastal compared to non-coastal respondents (Table 3). 

Taking results from each of the 5 years into account, the GH data suggest that there may be a slight health disadvantage for living in a coastal city, while the unhealthy days information indicates a slight trend toward fewer physically and mentally unhealthy days in the coastal MMSAs. Inspection of Figure 2, Figure 3, Figure 4, Figure 5, Figure 6 and Figure 7 reveals slight trends over the five-year period toward increasing mean percentages of zero PUHDs and zero MUHDs and decreases in the ≥14 days PUHD and MUHD data among coastal MMSAs relative to non-coastal MMSAs, with little apparent trend in the PMUHD data for either coastal or non-coastal MMSAs. It appears that a positive health trend occurred for coastal MMSAs compared to non-coastal MMSAs toward the latter two years of the study (2016, 2017), as measured by the self-reported PUHD and MUHD metrics. 

### 3.2. Comparing Health Responses for the Gulf Region Only: Gulf Coastal vs. Gulf Non-Coastal MMSAs 

For GH, only one result in the E, VG, G or F categories was significant, while the Gulf coastal MMSA residents responded P at a significantly higher rate in 3 of 5 years (2013, 2016, 2017) compared to those from non-coastal Gulf MMSAs (Table 4). Looking at the combined categories, Gulf coastal MMSA respondents reported poorer GH for 2 years (2013, 2016), with a lower proportion of respondents in the E/VG/G binning and a higher proportion in the F/P grouping. This indication of poorer health in the Gulf coastal MMSAs was supported by the unhealthy days data (Figure 2, Figure 3, Figure 4, Figure 5, Figure 6 and Figure 7). The coastal PUHDs showed a significantly lower proportion of responses in the 0-days category in 2 of 5 years (2013, 2014) and significantly higher responses in the ≥14 unhealthy days category in 4 of 5 years. The MUHD data showed a similar trend for Gulf coastal MMSAs, with significantly lower percentages in the 0-day category for 2 years (2013, 2015), and significantly higher in the ≥14-day category for those years as well as 2014. The PMUHD data revealed no significantly better results in the 0-day category for coastal in any year, with 2016 indicating significantly poorer health. Four of 5 years showed significantly higher (worse) responses in the ≥14-days category for the coastal respondents. Similarly, in comparing mean numbers of unhealthy days, the Gulf coastal MMSAs reported higher numbers of PUHDs in all years, MUHDs in 3 of 5 years (2013, 2014, 2015), and PMHUDs in all years. The overall mean numbers of unhealthy days were significantly higher for the coastal MMSAs in the PUHD, MUHD, and PMUHD categories, averaging 0.58–1.16 more unhealthy days for the Gulf coastal vs. Gulf non-coastal (Table 3). 

Inspection of the Gulf columns in Figure 2, Figure 3, Figure 4, Figure 5, Figure 6 and Figure 7 reveals a trend of increasing percentages of zero PUHDs among coastal MMSAs relative to non-coastal over the 5 years. No trends were observed in the ≥14 PUHD data, while the MUHD data indicated possible trends toward decreasing percentages of zero and increasing >14-days for the non-coastal compared to the coastal MMSAs. Similarly, there was a slight trend toward increasing percentages of ≥14-day PMUHDs among Gulf coastal MMSAs relative to non-coastal, with no clear trend for the 0-days PMUHD category.

Considering all the data, it appears that the self-reported health of residents of Gulf coastal MMSAs was significantly poorer than for those from non-coastal Gulf MMSAs.

### 3.3. Comparing Health Responses for Gulf Coastal vs. Non-Gulf Coastal MMSAs 

The proportion of GH reports in the E category from residents of Gulf coastal MMSAs was significantly lower compared to other coastal MMSAs in 1 year (2013). However, it was striking that in the P category, Gulf coastal MMSA reports were significantly higher (worse) in 4 of 5 years. In the complementary E/VG/G and F/P combined categories, we found significant differences in 4 of 5 years, with the Gulf coastal MMSAs reporting significantly lower percentages of E/VG/G health and significantly higher proportion of F/P health. These data indicate a negative GH ranking for Gulf coastal MMSA residence compared to living in a coastal MMSA elsewhere in the country (Table 5). 

There were no significant differences in reported PUHDs between residents of the Gulf coastal MMSAs and other coastal MMSAs in the 0-day category. However, in the ≥14-day category, the Gulf coastal MMSAs reported significantly higher proportions in 4 of 5 years. For MUHDs, the Gulf coastal MMSA data were significantly higher (better) in 1 year (2016) in the 0-day category, while they were significantly worse (higher) in 2 of 5 years (2013, 2015) in the ≥14-day group. For PMUHDs, differences were NS in all years in the 0-day category, but the Gulf coastal MMSA results were significantly higher (worse) in all 5 years in the ≥14-day category. 

The mean unhealthy days data indicated significantly poorer health for the Gulf coastal compared to the non-Gulf coastal MMSAs in 4 of 5 years for PUHDs, 2 of 5 years for MUHDs, and all 5 years for PMUHDs (Table 5, Figure 2, Figure 3, Figure 4, Figure 5, Figure 6 and Figure 7). Differences in overall means ranged from 0.42–1.26 unhealthy days (Table 3). 

### 3.4. Effect of Gulf MMSAs on the Coastal vs. Non-Coastal Comparison 

With the weight of evidence suggesting the self-reported health of residents in Gulf coastal MMSAs is significantly lower than other coastal MMSAs as well as non-coastal MMSAs in the Gulf, all Gulf MMSAs (coastal and non-coastal) were removed and coastal vs. non-coastal analyses rerun. The purpose was to determine if self-reported health differences would be altered by excluding the Gulf region from the analysis. Removal of all Gulf MMSAs generally resulted in slightly improved health responses for the coastal MMSAs relative to non-coastal, which in some cases changed the significance of those comparisons (Table 6). For GH, the formerly significant findings of higher GH in non-coastal MMSAs for 2016 (using the E/VG/G and F/P groupings) became NS. Similarly, for PUHDs, the previously significantly higher non-coastal proportion in the 0-day category became NS for 2014 and 2015. The 0 days category for 2017, which was previously NS, became statistically significant in this new analysis with a higher proportion of coastal respondents choosing that category, the opposite of the trend of any significant result from the previous analysis. Additionally, this analysis resulted in a newly significant greater proportion of non-coastal respondents in the ≥14-day category for the year 2016, extending the findings from 2017 to the previous year. Significant PUHD results from this non-Gulf analysis indicate a positive health status for coastal compared to non-coastal respondents, with the only exception being year 2013 where the proportion of 0-days responses was significantly higher for non-coastal.

For MUHDs, the new analysis rendered the previously significant greater proportion of non-coastal respondents in the 0 days category NS for years 2013 and 2015, while the year 2017 resulted in a newly significant greater proportion of coastal residents in the 0 days category. While results from the initial coastal vs. non-coastal analysis yielded mixed results for MUHDs, statistically significant MUHD results of this assessment indicate only a comparatively positive health status associated with coastal MMSAs. With PMUHDs, this non-Gulf analysis resulted in newly significant relationships for the years 2013 and 2014, where higher proportions of non-coastal respondents were in the ≥14-day category. Again, all statistically significant differences in PMUHDs generated by this non-Gulf assessment indicate only a comparatively positive health status associated with coastal MMSAs. The cumulative means from this analysis for PUHDs, MUHDs, and PMUHDS all were significantly lower (better) for coastal than non-coastal, with PUHD newly significant in this non-Gulf assessment (Table 3).

Overall, for GH, exclusion of the Gulf region MMSAs resulted in coastal having significantly better E scores for all years instead of 3 years as in the previous analysis. In the combined E/VG/G and F/P groupings, coastal was significantly worse in 2 years instead of 3 years in each category. The unhealthy days data showed a stronger positive effect for coastal without the Gulf, with improvements in PUHDs, MUHDs, and especially PMUHDs for coastal compared to non-coastal MMSAs. The unhealthy days data indicate better health, or at least fewer physically and mentally unhealthy days, in the non-Gulf coastal MMSAs, although the actual magnitude of the differences was relatively small (0.13–0.28 day) (see last two columns in Table 3 and Figure 2, Figure 3, Figure 4, Figure 5, Figure 6 and Figure 7).

Taking all results into account, the evidence suggests that living in any U.S. coastal urban area where at least 50% of the population resides within 20 km of a coastline is neither significantly better nor worse than residence in a non-coastal urban area. Since numerous other studies [8] have found some measures of better self-reported health for coastal compared to inland residents, this finding may be a result of inability of the data to reveal fine scale effects or other reasons including the up to 20-km distance from the coast included in our definition of a coastal MMSA. Closer proximity to coasts, particularly within a few km, blue space views, and visits to shoreline areas are associated with more and stronger positive health responses [7,9,50,51,52], including for older people [53,54]. Hooyberg et al. [7] reported better GH among Belgians residing within 5 km of a coast, but they were unable to discern significant differences at distances from the coast > 5 km. Similar results were reported by Garret et al. [4] for those within 5 km of a coast and no health effect for those at distances > 50 km inland. Garrett et al. [4] saw the greatest reduction in common mental disorders for economically disadvantaged people living within 1 km of a coast. Alternatively, our results may be indicative of circumstances where there are no differences in health between coastal and non-coastal location. For example, the GSPS study that was undertaken in the Gulf after the Deepwater Horizon oil spill found no differences in self-reported heath data between coastal and non-coastal areas [21,22,23]. Thus, results of previous research may not be generalizable to all coastal locations. Most importantly, it is clear that the overall results were affected by inclusion of the Gulf coastal MMSAs. Significant negative effects of coastal location were observed for the Gulf of Mexico coastal MMSAs. GH ratings were poorer and numbers of PUHDs, MUHDs, and PMUHDs experienced were higher for Gulf urban residents when compared to all other coastal or non-coastal subpopulations in the study. The magnitude of the differences was considerable, with ~0.4–1.3 days more of unhealthy physical, mental, and physical or mental condition for the Gulf respondents. When all Gulf MMSAs were removed from the analysis, a modest but important health advantage was observed for non-Gulf coastal locations compared to non-coastal areas. This finding supports the positive coastal health effect reported by previous researchers and suggests that potential health-promoting effects of coastal location may have been dampened in the Gulf region. We speculate that such dampening may be the result of the Gulf region’s history of major environmental disasters [16,17], myriad socioeconomic and health disparities [55], regular and increasing occurrence of harmful algal blooms [56], lack or inadequacy of health insurance [47], or other factors.

The environmental status of coastal areas should receive more attention in future studies, particularly as impacts of climate change such as increases in coastal storms, flooding, harmful algal blooms, water- and food-borne infectious diseases, and other problems arise [15]. Mishra et al. [57] provide an elegant BlueHealth Environment Assessment Tool for evaluating environmental characteristics of coastal areas and how they may affect health promoting effects and use of blue spaces. However, water quality and ecological factors are not yet included, although the authors indicate that a separate tool is being developed to assess these characteristics.

Future studies with more granular data may show a stronger positive coastal health effect, particularly if data can be obtained from longitudinal rather than cross-sectional studies [9,58] and for residential location closer to coastlines. It may also be important to consider timing of data collection, i.e., whether data were collected during or immediately following times when environmental and/or socio-economic factors were favorable or at least not negative, versus times of occurrences of periodic health-threatening problems common to some coastal areas, such as cyclones, harmful algal blooms, and flooding [16,18,20].

### 3.5. Demographic Characteristics

Associations between sex/gender, age, race/ethnicity, education, income, and other demographic factors with BRFSS health metrics have been explored in numerous previous studies, e.g., see [22,25,29,36,39,40]. Because our primary focus was on the comparison of coastal vs. non-coastal urban residency, and due to the previously mentioned concerns about use of linear regression approaches with these data, we did not include demographic characteristics as key variables. Future studies might examine the use of other regression methods [49] or other approaches that we were unable to incorporate here. Nonetheless, the 5 years of data provide some interesting information about health of residents of urban areas across the continental U.S. and in coastal and non-coastal locations. Overall, our results were generally in line with those of previous studies. 

Considering all 165 MMSA populations combined, approximately 52% of the respondents were female and 48% male. Overall, men reported significantly better general, physical and mental health than did women, with men reporting an average of 0.7 less physically and 1.1 less mentally unhealthy days respectively than women (Table 7). 

For race/ethnicity, we compared two categories, non-Hispanic White and non-White or Hispanic (hereafter referred to as white and non-white). Overall, for GH, whites reported significantly higher levels of E/VG/G combined and significantly lower levels of F/P than non-whites. However, looking at PUHDs and MUHDs, although the differences were small, a significantly higher proportion of non-whites than whites reported 0 unhealthy days, and there were no significant differences in the ≥14-days category or in PMUHDs. Also, no significant differences in mean numbers of PUHDs, MUHDs, or PMUHDs were discerned (Table 7). Overall, we found little difference in self-reported health metrics between whites and non-whites.

Trends for health reports with age, education, and income categories were as expected (Table 7). The percentages reporting E/VG/G health declined as age increased, while percentages reporting F/P health increased. Similar but less pronounced trends were observed for PUHD, but the opposite was seen for MUHD, with the percentage reporting 0 MUHDs increasing with age while the numbers reporting ≥14 MUHDs decreased. Considering PMUHDs, 0-day reports stayed nearly the same over age groups, while the ≥14-day numbers increased with age. Looking at the mean numbers of days reported, the PUHDs increased from 2.1 to 5.7 from the 18–24 age group to the >80 years age group, while numbers of MUHDs decreased from 4.5 to 2.2 days across those age groups. Thus, while physical infirmities increased with age, mental health issues appeared to decrease. These results reflect previous findings and may represent less stress from more settled lives among older people in contrast to more anxiety among those of younger age related to risky behaviors, relationship issues, work, financial, or other concerns [22,29]. 

As anticipated, education level trended positively alongside reported health metrics, with E/VG/G percentages increasing and F/P percentages decreasing with rising educational attainment. Results were not clear cut in the 0-day category for unhealthy days but were substantially lower in the ≥14 days category for PUHDs, MUHDs, and PMUHDs at the highest education level (college graduate) than for lower levels of education. The mean numbers of unhealthy days experienced reflected the same trend, with the numbers of unhealthy days reported by college graduates being starkly lower than each other category. 

Similarly, income levels were directly related to health results, with increasing percentages of E/VG/G and decreasing percentages of F/P reports with rising income, with marked differences between higher and lower income levels. For those in the highest category (>$75,000), ~94% reported E/VG/G health and only 6% F/P. The unhealthy days data showed the same trend even more strongly, with mean numbers of PUHDs, MUHDs, and PMUHDs decreasing from 7.5, 7.4, and 8.9 days, respectively at the lowest income level to 2.1, 2.4, and 2.6 days, respectively at the highest. 

Comparisons of demographic characteristics between coastal and non-coastal MMSAs are summarized in Table 8. Unsurprisingly, white respondents comprised a significantly larger proportion of populations in both coastal and non-coastal MMSAs compared to non-whites, averaged over the five years of the study. However, all coastal MMSAs tended to be more racially diverse than all non-coastal, averaging ~48% non-white in coastal compared to ~36% in the non-coastal MMSA population. We found no significant differences in proportions of males and females between coastal and non-coastal MMSAs in any of the years evaluated, although there were slightly more females than males in all years (~52% vs. 48%). Age compositions of the respective populations were similar, but percentages of young (18–24 y) people were significantly lower in coastal compared to non-coastal MMSAs, while proportions of seniors aged 65–69, 75–79 and >80 were significantly higher in the coastal MMSAs every year, although they composed <7% of the populations. For the >80 group, the difference was significant in every year, not just the overall mean. The higher proportion of older people in coastal areas may reflect the relative attractiveness of coastal location for retirees. Some differences in education and income were also noted. Coastal populations had significantly less people with only a high school education or equivalent or with only 1–3 years of college or technical education compared to those in non-coastal MMSAs, with significantly greater percentages of those with a college degree or more in coastal MMSAs. Coastal respondents reported higher percentages at both of the lower (<$10,00 and <$15,000) and highest (>$75,000) ends of the income scale, with non-coastal respondents reporting higher percentages for the next highest income levels (<$50,000 and <$75,000). 

The Gulf coast MMSAs were less racially diverse than non-coastal Gulf MMSAs, with non-whites making up only about 35% of Gulf coastal MMSA populations on average compared to ~51% in Gulf non-coastal MMSAs. They were also less diverse than the all-coastal category (47% non-white) but racial proportions were similar to those in the all-non-coastal MMSA group. Removing the Gulf coast MMSAs from the coastal grouping resulted only in a slight (1%) change in the relative proportions of white and non-white respondents in the remaining population. Age structures were relatively similar, although the Gulf coast MMSAs tended to have fewer young adults (ages 18–24), while proportions of seniors (ages 65–69, 70–74, 75–79, and >80) were consistently higher than in the Gulf non-coastal MMSAs and non-Gulf coastal. Regarding education, Gulf coast MMSAs had consistently higher proportions with a high school diploma or equivalent, but lower percentages of college graduates compared to Gulf non-coastal. Few significant differences in income levels were noted, except at the highest level, >$75,000, where Gulf coastal respondent reports were lower on average. Comparing Gulf coastal to other (non-Gulf) coastal MMSAs, the Gulf respondents reflected larger proportions of older people and lower percentages of college graduates and in the highest income group. When the Gulf region MMSAs were excluded from the analysis, the remaining (non-Gulf) coastal MMSAs had a higher proportion of non-whites, >80 year-old residents, college graduates, and people in the highest income group compared to non-Gulf non-coastal MMSAs (Table 8). 

While we did not explore relationships between demographic factors and health in detail, findings relating poorer health to female gender, non-white ethnicity, and lower education and income levels have been reported in numerous previous studies, e.g., [29,36,59]. Here, coastal MMSAs in general had higher proportions of non-whites, but Gulf coastal MMSAs were less diverse (higher proportions of whites) and more comparable to ethnic proportions of non-coastal areas. Based on race-health associations [29,36], one might expect that the Gulf coastal MMSAs with their higher proportion of white populations would have reported better health, yet such was not the case. This likely reflects the absence of any marked difference in overall health responses from whites and non-whites. Educational achievement, which is often associated with better health, in general tended to be higher for coastal respondents, but this was not the case for the Gulf MMSAs. 

## 4. Conclusions

Notwithstanding limitations of the data, our results suggest that even at the level of large urban areas, one can distinguish some health effects that may be associated with coastal location. By itself, residence in a *non-Gulf of Mexico U.S. coastal urban area* in which >50% of the population lives within 20 km of a coast appears to be associated with modestly better self-reported health than residence in non-coastal urban areas. That we can perceive an effect even at the scale of the BRFSS SMART data points to the apparent robustness of the association between coastal exposure and better health. Equally important, our analyses revealed that self-reported health of urban residents in the disaster-prone Gulf coast region appears to be significantly poorer than that of residents from other urban areas, both coastal and non-coastal. Although concerning, the Gulf results in particular should be considered preliminary, due to the relatively small number of MMSAs from which data were available in this region. We suggest that the comparative health of residents of this region should receive more in-depth assessment in the near future. The temporal changes observed across the time horizon of this study support the need for longitudinal or at least multi-year monitoring. While BRFSS data can be useful for a variety of health analyses, they are not sufficiently detailed to allow determination of mechanisms of action or to attribute effects to a specific cause, e.g., an environmental disaster. This weakness underscores the need for much more clinical data from longitudinal studies [60] such as the large, continuously operating longitudinal cohort studies proposed for a Gulf of Mexico Community Health Observing System [16,17], in addition to existing cross-sectional studies.

## Figures and Tables

**Table 1:** Summary of types and abbreviations of self-reported responses to the BRFSS Healthy Days questions.

General health (GH) ratings: reported as % of days over the entire previous 30-day period	
E	Excellent
VG	Very Good
G	Good
F	Fair
P	Poor
E/VG/G	Excellent/Very Good/Good combined
F/P	Fair/Poor combined
Unhealthy Days (% or number of days over the last 30 days, grouped as 0 days, 1–13 days, ≥14 days)	
PUHDs	Physically Unhealthy Days
MUHDs	Mentally Unhealthy Days
PMUHDs	Physically or Mentally Unhealthy Days (refers not only to unhealthy days but more specifically to days when poor physical or mental health interfered with conduct of daily activities)

**Table 2:** Comparison of all coastal vs. all non-coastal MMSAs: Mean self-rated general health and unhealthy days. (See table notes for explanation).

		Self-Rated General Health (%)							PUHD (%)		MUHD (%)		PMUHD (%)		PUHD	MUHD	PMUHD
Year	Location	E	VG	G	F	P	E/VG/G	F/P	0 Days	≥14 Days	0 Days	≥14 Days	0 Days	≥14 Days	Mean Days	Mean Days	Mean Days
2013	Coastal	**20.2 (0.6)**	**31.4 (0.7)**	30.7 (0.7)	**13.3 (0.5)**	4.3 (0.3)	**82.4 (0.5)**	**17.6 (0.5)**	**63.1 (0.7)**	11.4 (0.5)	**64.6 (0.7)**	11.4 (0.5)	57.4 (0.9)	13.9 (0.7)	3.8 (0.11)	3.72 (0.11)	**4.46 (** **0.16** **)**
	Non-Coastal	**19.1 (0.4)**	**33.3 (0.5)**	31 (0.4)	**12.2 (0.3)**	4.4 (0.2)	**83.4 (0.4)**	**16.6 (0.3)**	**65.2 (0.4)**	11.3 (0.3)	**66.5 (0.4)**	11.1 (0.3)	58.1 (0.7)	15 (0.5)	3.69 (0.08)	3.61 (0.07)	**4.69 (0.12)**
2014	Coastal	20.4 (0.5)	**31.3 (0.7)**	30.5 (0.7)	**13.5 (0.5)**	4.3 (0.4)	**82.2 (0.6)**	**17.8 (0.6)**	**63.8 (0.7)**	11.6 (0.5)	65.4 (0.7)	10.9 (0.4)	58.1 (1.0)	14.1 (0.7)	3.79 (0.12)	3.54 (0.11)	4.51 (0.20)
	Non-Coastal	19.5 (0.4)	**33 (0.4)**	31 (0.4)	**12 (0.4)**	4.5 (0.2)	**83.5 (0.3)**	**16.5 (0.4)**	**65.8 (0.4)**	11.3 (0.3)	66.4 (0.4)	11.1 (0.3)	58.5 (0.7)	14.9 (0.5)	3.67 (0.08)	3.59 (0.08)	4.65 (0.10)
2015	Coastal	20.2 (0.5)	**32.2 (0.7)**	31.1 (0.6)	12.4 (0.5)	4.1 (0.3)	83.5 (0.5)	16.5 (0.5)	**63.6 (0.7)**	11.2 (0.4)	**64.6 (0.7)**	11.3 (0.5)	57.1 (0.9)	14.1 (0.7)	3.71 (0.11)	3.65 (0.11)	4.51 (0.20)
	Non-Coastal	19.5 (0.4)	**33 (0.5)**	31.5 (0.5)	12 (0.3)	4.1 (0.2)	83.9 (0.4)	16.1 (0.4)	**65.1 (0.5)**	11.1 (0.3)	**66.2 (0.5)**	10.8 (0.3)	58.4 (0.7)	14.4 (0.5)	3.64 (0.08)	3.54 (0.08)	4.52 (0.10)
2016	Coastal	**20.5 (0.5)**	**31.5 (0.6)**	30.8 (0.6)	**13.1 (0.4)**	4.1 (0.3)	**82.8 (0.5)**	**17.2 (0.5)**	65.3 (0.6)	11 (0.4)	**66.1 (0.6)**	**10.5 (0.4)**	57.4 (0.9)	14.2 (0.6)	3.62 (0.10)	**3.48 (0.10)**	4.51 (0.20)
	Non-Coastal	**19.3 (0.4)**	**33.2 (0.5)**	31.3 (0.5)	**12 (0.3)**	4.3 (0.2)	**83.8 (0.4)**	**16.2 (0.4)**	65.4 (0.5)	11.4 (0.4)	**65.1 (0.5)**	**11.5 (0.3)**	57.8 (0.7)	14.7 (0.5)	3.71 (0.08)	**3.74 (0.08)**	4.62 (0.12)
2017	Coastal	**19.7 (0.7)**	31.8 (0.7)	31.5 (0.7)	12.9 (0.5)	4.1 (0.3)	83 (0.5)	17 (0.6)	65 (0.7)	**10.7 (0.5)**	64.7 (0.7)	**11.3 (0.4)**	56.6 (1.0)	**13.9 (0.7)**	3.61 (0.12)	**3.7 (0.12)**	4.51 (0.16)
	Non-Coastal	**18.4 (0.4)**	32.2 (0.5)	32.1 (0.5)	12.9 (0.4)	4.4 (0.2)	82.7 (0.4)	17.3 (0.4)	64.7 (0.5)	**11.7 (0.4)**	63.9 (0.5)	**12.2 (0.4)**	56.7 (0.7)	**15.2 (0.6)**	3.78 (0.09)	**3.94 (0.09)**	4.73 (0.13)

Data are expressed in mean percentages of respondents or mean numbers of days with 95% confidence limits expressed as a single + number in parentheses beside the mean. Means in bold type are significantly different at *p* < 0.05. Abbreviations: PUHD = physically unhealthy days; MUHD = mentally unhealthy days; PMUHD = physically or mentally unhealthy days; E = excellent; VG = very good; G = good; F = fair; P = poor; E/VG/G = excellent, very good, and good combined; F/P = fair and poor combined, with alternating gray and white backgrounds to separate paired comparisons.

**Table 3:** Overall (average over all years) numbers of unhealthy days. (See table notes for explanation).

	C	NC	GC	GNC	GC	NGC	NGC	NGNC
PUHD	3.71 (0.05)	3.70 (0.04)	**4.40** (0.16)	**3.46** (0.10)	**4.40** (0.16)	**3.65** (0.05)	**3.65** (0.05)	**3.78** (0.03)
MUHD	**3.62** (0.05)	**3.69** (0.03)	**4.01** (0.16)	**3.43** (0.10)	**4.01** (0.16)	**3.59** (0.05)	**3.59** (0.05)	**3.77** (0.04)
PMUHD	**4.50** (0.07)	**4.64** (0.06)	**5.67** (0.25)	**4.51** (0.15)	**5.67** (0.25)	**4.41** (0.07)	**4.41** (0.07)	**4.69** (0.05)

Numbers in bold type are significantly different at *p* < 0.05. Numbers in parentheses are 95% confidence intervals expressed as a single ± number in parentheses beside the mean. PUHD = physically unhealthy days, MUHD = mentally unhealthy days, PMUHD = physically or mentally unhealthy days. MMSA comparisons are all coastal (C) vs. all non-coastal NC); Gulf coastal (GC) vs. Gulf non-coastal (GNC); Gulf coastal (GC) vs. non-Gulf coastal (NGC); and non-Gulf coastal (NGC) vs. non-Gulf non-coastal (NGNC). For ease of reading, comparisons for significance are made in pairs: C vs. NC, GC vs. GNC, GC vs. NGC, and NGC vs. NGNC, with alternating gray and white backgrounds to separate paired comparisons.

**Table 4:** Comparison of all Gulf Coastal vs. Gulf Non-Coastal MMSAs: Mean self-rated general health and unhealthy days. (See table notes for explanation).

		Self-Rated General Health (%)														PUHD (%)				MUHD (%)				PMUHD (%)				PUHD		MUHD		PMUHD	
Year	Location	E		VG		G		F		P		E/VG/G		F/P		0 Days		>14 Days		0 Days		>14 Days		0 Days		>14 Days		Mean Days		Mean Days		Mean Days	
2013	Gulf Coastal	18.3	(1.4)	30.5	(1.7)	31.1	(1.7)	14.6	60.9	**(5.4)**	**(0.8)**	**80**	**(1.3)**	**20**	**(1.4)**	**60.9**	**(1.8)**	**14.1**	**(1.2)**	**64.7**	**(1.7)**	**13.8**	**(1.4)**	57.3	(2.4)	**14.1**	**(1.2)**	**4.53**	**(0.31)**	**4.33**	**(0.31)**	**5.31**	**(0.44)**
	Non-Gulf Coastal	19.1	(1.1)	30.2	(1.1)	33.1	(1.3)	13.3	67	**(4.3)**	**(0.5)**	**82.4**	**(1.0)**	**17.6**	**(1.0)**	**67**	**(1.3)**	**10.6**	**(0.8)**	**69.6**	**(1.2)**	**10.3**	**(0.8)**	59.2	(1.8)	**10.6**	**(0.8)**	**3.45**	**(0.19)**	**3.32**	**(0.19)**	**4.49**	**(0.31)**
2014	Gulf Coastal	19.5	(1.9)	30.3	(2.0)	31.1	(2.2)	14.3	62.7	(4.7)	(1.0)	80.9	(1.7)	19.1	(1.8)	**62.7**	**(2.2)**	**12.6**	**(1.4)**	67	(2.1)	**12.2**	**(1.6)**	55.1	(3.0)	12.6	(1.4)	**4.15**	**(0.35)**	**3.82**	**(0.36)**	**5.34**	**(0.55)**
	Non-Gulf Coastal	19.5	(1.0)	30.2	(1.1)	32.6	(1.2)	12.9	68.1	(4.8)	(0.6)	82.3	(0.9)	17.7	(1.0)	**68.1**	**(1.1)**	**10.4**	**(0.7)**	68.4	(1.1)	**10.2**	**(0.8)**	59.5	(1.7)	10.4	(0.7)	**3.41**	**(0.18)**	**3.3**	**(0.18)**	**4.57**	**(0.29)**
2015	Gulf Coastal	18	(2.8)	29.9	(2.2)	31.3	(2.2)	13.7	62.8	(6.3)	(1.2)	80	(1.8)	20	(1.9)	62.8	(2.2)	**14.8**	**(1.7)**	65.2	(2.3)	**13.1**	**(1.7)**	53.9	(3.3)	**14.8**	**(1.7)**	**4.67**	**(0.41)**	**4.16**	**(0.41)**	**6.04**	**(0.62)**
	Non-Gulf Coastal	18.9	(1.5)	28.8	(1.5)	34.2	(1.7)	13.2	65.5	(6.9)	(0.8)	81.9	(1.4)	18.1	(1.3)	65.5	(1.6)	**10.9**	**(1.1)**	69.3	(1.6)	**10**	**(1.1)**	57.9	(2.3)	**10.9**	**(1.1)**	**3.58**	**(0.26)**	**3.23**	**(0.27)**	**4.50**	**(0.37)**
2016	Gulf Coastal	19.1	(1.5)	31.7	(1.7)	29.4	(1.7)	13.7	64.5	**(6.1)**	**(0.9)**	**80.2**	**(1.4)**	**19.8**	**(1.6)**	64.5	(1.7)	**13.3**	**(1.2)**	68	(1.7)	12	(1.2)	**54.2**	**(2.6)**	**13.3**	**(1.2)**	**4.25**	**(0.31)**	3.74	(0.30)	**5.77**	**(0.50)**
	Non-Gulf Coastal	19.9	(1.3)	30.9	(1.5)	32.5	(1.5)	12.5	67.4	**(4.2)**	**(0.6)**	**83.3**	**(1.2)**	16.7	(1.2)	67.4	(1.4)	**10.5**	**(1.0)**	67	(1.4)	10.8	(1.0)	**60.4**	**(2.0)**	**10.5**	**(1.0)**	**3.41**	**(0.23)**	3.51	(0.24)	**4.34**	**(0.33)**
2017	Gulf Coastal	19.4	(2.0)	30.5	(2.1)	**29.9**	**(2.2)**	13.6	65	**(6.6)**	**(1.1)**	79.8	(1.7)	20.2	(1.8)	65	(2.1)	13.6	(1.5)	65.9	(2.1)	12.9	(1.5)	54.7	(3.1)	**13.6**	**(1.5)**	**4.43**	**(0.40)**	4.04	(0.37)	**5.93**	**(0.60)**
	Non-Gulf Coastal	18.7	(1.3)	28.7	(1.3)	**34.2**	**(1.6)**	14.1	66.7	**(4.3)**	**(0.7)**	81.6	(1.2)	18.4	(1.3)	66.7	(1.4)	11	(1.1)	65.7	(1.5)	11.7	(1.0)	56.6	(2.2)	**11**	**(1.1)**	**3.59**	**(0.25)**	3.79	(0.25)	**4.71**	**(0.39)**

Data are expressed in mean percentages of respondents or mean numbers of days with 95% confidence limits expressed as a single + number in parentheses beside the mean. Means in bold type are significantly different at *p* < 0.05. Abbreviations are as follows: PUHD = physically unhealthy days; MUHD = mentally unhealthy days; PMUHD = physically or mentally unhealthy days; E = excellent; VG = very good; G = good; F = fair; P = poor; E/VG/G = excellent, very good, and good combined; F/P = fair and poor combined, with alternating gray and white backgrounds to separate paired comparisons.

**Table 5:** Comparison of Gulf Coastal vs. Non-Gulf Coastal MMSAs: Mean self-rated general health and unhealthy days. (See table notes for explanation).

		Self-Rated General Health (%)														PUHD (%)				MUHD (%)				PMUHD (%)				PUHD		MUHD		PMUHD	
Year	Location	E		VG		G		F		P		E/VG/G		F/P		0 Days		>14 Days		0 Days		>14 Days		0 Days		>14 Days		Mean Days		Mean Days		Mean Days	
2013	Gulf Coastal	**18.3**	**(1.4)**	30.5	(1.7)	31.1	(1.7)	14.6	(1.2)	**5.4**	**(0.8)**	**80**	**(1.3)**	**20**	**(1.4)**	60.9	(1.8)	**14.1**	**(1.2)**	64.7	(1.7)	**13.8**	**(1.4)**	57.3	(2.4)	**17.5**	**(1.9)**	**4.53**	**(0.31)**	**4.33**	**(0.31)**	**5.31**	**(0.44)**
	Non-Gulf Coastal	**20.4**	**(0.6)**	31.5	(0.7)	30.7	(0.7)	13.2	(0.5)	**4.2**	**(0.3)**	**82.6**	**(0.6)**	**17.4**	**(0.6)**	63.3	(0.7)	**11.2**	**(0.5)**	64.6	(0.7)	**11.2**	**(0.5)**	57.4	(1.0)	**13.6**	**(0.7)**	**3.74**	**(0.12)**	**3.67**	**(0.11)**	**4.39**	**(0.17)**
2014	Gulf Coastal	19.5	(1.9)	30.3	(2.0)	31.1	(2.2)	14.3	(1.7)	4.7	(1.0)	80.9	(1.7)	19.1	(1.8)	62.7	(2.2)	12.6	(1.4)	67	(2.1)	12.2	(1.6)	55.1	(3.0)	**16.7**	**(2.4)**	4.15	(0.35)	3.82	(0.36)	**5.34**	**(0.55)**
	Non-Gulf Coastal	20.4	(0.6)	31.4	(0.7)	30.4	(0.8)	13.4	(0.6)	4.3	(0.3)	82.3	(0.6)	17.7	(0.6)	63.9	(0.8)	11.5	(0.5)	65.7	(0.5)	11.4	(0.3)	58.3	(1.1)	**13.9**	**(0.7)**	3.77	(0.12)	3.52	(0.12)	**4.45**	**(0.18)**
2015	Gulf Coastal	18	(2.8)	29.9	(2.2)	31.3	(2.2)	13.7	(1.7)	**6.3**	**(1.2)**	**80**	**(1.8)**	**20**	**(1.9)**	62.8	(2.2)	**14.8**	**(1.7)**	65.2	(2.3)	**13.1**	**(1.7)**	53.9	(3.3)	**19.3**	**(2.5)**	**4.67**	**(0.41)**	**4.16**	**(0.41)**	**6.04**	**(0.62)**
	Non-Gulf Coastal	20.2	(0.6)	32.4	(0.6)	31	(0.7)	12.4	(0.4)	**3.9**	**(0.3)**	**83.7**	**(0.5)**	**16.3**	**(0.5)**	63.7	(0.7)	**10.9**	**(0.5)**	65.3	(0.4)	**11.2**	**(0.3)**	57.3	(1.0)	**13.8**	**(0.7)**	**3.65**	**(0.11)**	**3.62**	**(0.11)**	**4.41**	**(0.16)**
2016	Gulf Coastal	19.1	(1.5)	31.7	(1.7)	29.4	(1.7)	13.7	(1.4)	**6.1**	**(0.9)**	**80.2**	**(1.4)**	**19.8**	**(1.6)**	64.5	(1.7)	**13.3**	**(1.2)**	**68**	**(1.7)**	12	(1.2)	54.2	(2.6)	**18.1**	**(2.0)**	**4.25**	**(0.31)**	3.74	(0.30)	**5.77**	**(0.50)**
	Non-Gulf Coastal	20.6	(0.6)	31.5	(0.6)	30.9	(0.7)	13	(0.5)	**3.9**	**(0.3)**	**83.1**	**(0.5)**	**16.9**	**(0.6)**	65.4	(0.6)	**10.8**	**(0.4)**	**64.5**	**(0.4)**	11.7	(0.3)	57.7	(0.9)	**13.8**	**(0.7)**	**3.57**	**(0.10)**	3.46	(0.10)	**4.41**	**(0.15)**
2017	Gulf Coastal	19.4	(2.0)	30.5	(2.1)	29.9	(2.2)	13.6	(1.6)	**6.6**	**(1.1)**	**79.8**	**(1.7)**	**20.2**	**(1.8)**	65	(2.1)	**13.6**	**(1.5)**	65.9	(2.1)	12.9	(1.5)	54.7	(3.1)	**20.2**	**(2.6)**	**4.43**	**(0.40)**	4.04	(0.37)	**5.93**	**(0.60)**
	Non-Gulf Coastal	19.8	(0.6)	31.9	(0.8)	31.6	(0.7)	12.8	(0.5)	**3.9**	**(0.3)**	**83.3**	**(0.5)**	**16.7**	**(0.6)**	65	(0.7)	**10.5**	**(0.5)**	63.2	(0.5)	12.4	(0.4)	56.8	(1.0)	**13.4**	**(0.7)**	**3.54**	**(0.12)**	3.67	(0.12)	**4.39**	**(0.17)**

Data are expressed in mean percentages of respondents or mean numbers of days) with 95% confidence limits expressed as a single + number in parentheses beside the mean. Means in bold type are significantly different at *p* < 0.05. Abbreviations are as follows: PUHD = physically unhealthy days; MUHD = mentally unhealthy days; PMUHD = physically or mentally unhealthy days; E = excellent; VG = very good; G = good; F = fair; P = poor; E/VG/G = excellent, very good, and good combined; F/P = fair and poor combined, with alternating gray and white backgrounds to separate paired comparisons.

**Table 6:** Comparison of all non-Gulf coastal vs. all non-Gulf non-coastal MMSAs (all Gulf MMSAs excluded): Mean self-rated general health and unhealthy days. (See table notes for explanation).

		Self-Rated General Health (%)														PUHD (%)				MUHD (%)				PMUHD (%)				PUHD		MUHD		PMUHD	
Year	Location	E		VG		G		F		P		E/VG/G		F/P		0 Days		>14 Days		0 Days		>14 Days		0 Days		>14 Days		Mean Days		Mean Days		Mean Days	
2013	Coastal	**20.4**	**(0.6)**	**31.5**	**(0.7)**	30.7	(0.7)	**13.2**	**(0.5)**	4.2	(0.3)	**82.6**	**(0.6)**	**17.4**	**(0.6)**	**63.3**	**(0.7)**	11.2	(0.5)	64.6	(0.7)	11.2	(0.5)	57.4	(1.0)	**13.6**	**(0.7)**	3.74	(0.12)	3.67	(0.11)	**4.39**	**(0.17)**
	Non-Coastal	**19.1**	**(0.4)**	**34.4**	**(0.4)**	30.3	(0.4)	**11.8**	**(0.3)**	4.4	(0.2)	**83.8**	**(0.3)**	**16.2**	**(0.4)**	**64.6**	**(0.4)**	11.6	(0.3)	65.4	(0.5)	11.4	(0.3)	57.8	(0.7)	**15.2**	**(0.5)**	3.77	(0.08)	3.7	(0.08)	**4.75**	**(0.13)**
2014	Coastal	**20.4**	**(0.6)**	**31.4**	**(0.7)**	30.4	(0.8)	**13.4**	**(0.6)**	4.3	(0.3)	**82.3**	**(0.6)**	**17.7**	**(0.6)**	63.9	(0.8)	11.5	(0.5)	65.3	(0.7)	10.8	(0.5)	58.3	(1.1)	**13.9**	**(0.7)**	3.77	(0.12)	3.52	(0.12)	**4.45**	**(0.18)**
	Non-Coastal	**19.5**	**(0.4)**	**33.9**	**(0.5)**	30.5	(0.4)	**11.7**	**(0.4)**	4.4	(0.2)	**83.9**	**(0.3)**	**16**	**(0.5)**	65	(0.5)	11.5	(0.4)	65.7	(0.5)	11.4	(0.3)	58.2	(0.7)	**15.1**	**(0.5)**	3.76	(0.08)	3.69	(0.08)	**4.68**	**(0.12)**
2015	Coastal	**20.2**	**(0.6)**	**32.4**	**(0.6)**	31	(0.7)	12.4	(0.4)	3.9	(0.3)	83.7	(0.5)	16.3	(0.5)	63.7	(0.7)	10.9	(0.5)	64.6	(0.6)	11.2	(0.5)	57.3	(1.0)	13.8	(0.7)	3.65	(0.11)	3.62	(0.11)	4.41	(0.16)
	Non-Coastal	**19.4**	**(0.4)**	**34**	**(0.4)**	30.9	(0.4)	11.7	(0.4)	4	(0.2)	84.3	(0.3)	15.7	(0.4)	64.7	(0.4)	11.3	(0.3)	65.3	(0.4)	11.2	(0.3)	58.5	(0.6)	14.5	(0.5)	3.71	(0.07)	3.65	(0.08)	4.54	(0.12)
2016	Coastal	**20.6**	**(0.6)**	**31.5**	**(0.6)**	30.9	(0.7)	13	(0.5)	3.9	(0.3)	83.1	(0.5)	16.9	(0.6)	65.4	(0.6)	**10.8**	**(0.4)**	**65.9**	**(0.7)**	**10.4**	**(0.4)**	57.7	(0.9)	13.8	(0.7)	**3.57**	**(0.10)**	**3.46**	**(0.10)**	**4.41**	**(0.15)**
	Non-Coastal	**19**	**(0.4)**	**34**	**(0.4)**	30.9	(0.4)	11.8	(0.3)	4.3	(0.2)	83.9	(0.4)	16.1	(0.3)	64.8	(0.4)	**11.8**	**(0.3)**	**64.5**	**(0.4)**	**11.7**	**(0.3)**	57	(0.6)	15	(0.5)	**3.81**	**(0.07)**	**3.82**	**(0.08)**	**4.71**	**(0.12)**
2017	Coastal	**19.8**	**(0.6)**	**31.9**	**(0.8)**	31.6	(0.7)	12.8	(0.5)	3.9	(0.3)	83.3	(0.5)	16.7	(0.6)	**65**	**(0.7)**	**10.5**	**(0.5)**	**64.6**	**(0.7)**	**11.1**	**(0.5)**	56.8	(1.0)	**13.4**	**(0.7)**	**3.54**	**(0.12)**	**3.67**	**(0.12)**	**4.39**	**(0.17)**
	Non-Coastal	**18.3**	**(0.4)**	**33.4**	**(0.5)**	31.4	(0.4)	12.5	(0.3)	4.4	(0.2)	83.1	(0.4)	16.9	(0.4)	**64**	**(0.5)**	**12**	**(0.3)**	**63.2**	**(0.5)**	**12.4**	**(0.4)**	56.7	(0.6)	**15.1**	**(0.5)**	**3.85**	**(0.08)**	**4**	**(0.08)**	**4.74**	**(0.11)**

Data are expressed in mean percentages of respondents or mean numbers of days) with 95% confidence limits expressed as a single + number in parentheses beside the mean. Means in bold type are significantly different at *p* < 0.05. Abbreviations are as follows: PUHD = physically unhealthy days; MUHD = mentally unhealthy days; PMUHD = physically or mentally unhealthy days; E = excellent; VG = very good; G = good; F = fair; P = poor; E/VG/G = excellent, very good, and good combined; F/P = fair and poor combined, with alternating gray and white backgrounds to separate paired comparisons.

**Table 7:** Summary of general health and unhealthy days data by demographic categories for all MMSAs over all five years of the study. (See table notes for explanation).

	Self-Rated General Health (%)		PUHD (%)		MUHD (%)		PMUHD (%)		PUHD	MUHD	PMUHD
	E/VG/G	F/P	0 Days	≥14 Days	0 Days	≥14 Days	0 Days	≥14 Days	Mean Days	Mean Days	Mean Days
**Sex/Gender**											
Men	**84.1**	**15.9**	**68.0**	**10.1**	**70.1**	**9.5**	**59.0**	14.2	**3.3**	**3.1**	4.5
	**(0.2)**	**(0.2)**	**(0.2)**	**(0.1)**	**(0.3)**	**(0.1)**	**(0.3)**	(0.3)	**(0.1)**	**(0.0)**	(0.1)
Women	**82.3**	**17.7**	**61.8**	**12.4**	**60.9**	**12.9**	**56.6**	14.7	**4.0**	**4.2**	4.6
	**(0.2)**	**(0.2)**	**(0.3)**	**(0.2)**	**(0.3)**	**(0.1)**	**(0.4)**	(0.3)	**(0.1)**	**(0.0)**	(0.1)
**Race/Ethnicity**											
White	**86.0**	**14.0**	**64.2**	11.3	**65.0**	11.1	57.8	14.3	3.7	3.6	4.5
	**(0.1)**	**(0.2)**	**(0.1)**	(0.1)	**(0.2)**	(0.1)	(0.3)	(0.2)	(0.1)	(0.1)	(0.1)
Non-White	**79.2**	**20.8**	**65.7**	11.2	**65.8**	11.4	57.4	14.7	3.6	3.7	4.6
	**(0.3)**	**(0.3)**	**(0.4)**	(0.2)	**(0.4)**	(0.3)	(0.5)	(0.3)	(0.1)	(0.0)	(0.1)
**Age (Years)**											
18–24	91.6	8.4	66.6	5.1	51.9	13.6	60.8	7.0	2.1	4.5	2.7
	(0.4)	(0.3)	(0.6)	(0.2)	(0.6)	(0.4)	(0.8)	(0.4)	(0.1)	(0.1)	(0.1)
65–69	77.5	22.5	62.9	15.3	75.0	8.6	56.2	18.5	4.8	2.8	5.6
	(0.5)	(0.6)	(0.6)	(0.4)	(0.5)	(0.3)	(0.9)	(0.7)	(0.1)	(0.1)	(0.2)
70–74	77.1	22.9	63.4	15.5	78.6	7.1	58.2	18.5	4.8	2.3	5.6
	(0.6)	(0.6)	(0.7)	(0.5)	(0.6)	(0.4)	(1.0)	(0.8)	(0.1)	(0.1)	(0.2)
75–79	74.3	25.7	61.4	17.2	79.9	6.7	59.1	20.2	5.3	2.2	5.9
	(0.8)	(0.8)	(0.8)	(0.7)	(0.7)	(0.4)	(1.3)	(1.0)	(0.2)	(0.1)	(0.3)
80+	71.5	28.5	60.1	18.5	80.7	6.7	61.0	19.7	5.7	2.2	6.0
	(0.6)	(0.7)	(0.7)	(0.6)	(0.6)	(0.5)	(1.0)	(0.8)	(0.2)	(0.1)	(0.2)
**Education (Years)**											
1–8	53.6	46.4	58.2	20.8	67.1	14.9	52.7	23.8	6.2	4.5	7.0
	(1.2)	(1.1)	(1.1)	(0.9)	(1.1)	(0.8)	(1.5)	(1.3)	(0.2)	(0.2)	(0.3)
12	80.5	19.5	64.4	12.9	65.9	12.7	57.8	16.6	4.1	4.0	5.1
	(0.2)	(0.3)	(0.4)	(0.2)	(0.4)	(0.3)	(0.5)	(0.4)	(0.1)	(0.1)	(0.1)
15	85.1	14.9	63.5	11.5	62.9	12.2	57.1	14.4	3.8	4.0	4.6
	(0.3)	(0.2)	(0.3)	(0.2)	(0.4)	(0.3)	(0.5)	(0.3)	(0.0)	(0.0)	(0.0)
16	92.9	7.1	69.5	6.1	68.3	6.6	61.1	7.9	2.3	2.5	2.9
	(0.1)	(0.2)	(0.2)	(0.1)	(0.3)	(0.2)	(0.4)	(0.2)	(0.0)	(0.0)	(0.1)
**Income** **($ × 10^3^)**											
<10	61.7	38.3	49.2	25.2	49.6	25.0	40.8	30.6	7.5	7.4	8.9
	(0.9)	(1.0)	(1.0)	(0.9)	(1.0)	(0.9)	(1.2)	(1.0)	(0.2)	(0.2)	(0.2)
<15	61.6	38.4	48.8	25.1	54.6	21.2	43.7	28.8	7.5	6.3	8.4
	(0.9)	(0.9)	(0.9)	(0.8)	(1.0)	(0.8)	(1.2)	(1.0)	(0.2)	(0.2)	(0.2)
<50	85.2	14.8	66.1	10.2	65.9	10.4	59.7	12.2	3.4	3.5	4.0
	(0.4)	(0.4)	(0.5)	(0.3)	(0.5)	(0.3)	(0.9)	(0.5)	(0.1)	(0.1)	(0.1)
<75	88.9	11.1	67.5	8.3	66.3	9.2	61.1	10.0	2.9	3.2	3.4
	(0.3)	(0.3)	(0.5)	(0.2)	(0.4)	(0.3)	(0.6)	(0.4)	(0.1)	(0.0)	(0.1)
>75	93.9	6.1	71.0	5.4	69.7	6.5	63.6	6.8	2.1	2.4	2.6
	(0.1)	(0.2)	(0.3)	(0.2)	(0.3)	(0.1)	(0.5)	(0.2)	(0.0)	(0.0)	(0.0)

Data represent overall means and 95% confidence limits (expressed as a single + number in parentheses). Numbers in bold type are significantly differentat *p* < 0.05. Education years are: 1–8 = elementary school; 12 = high school or equivalent; 15 = 1–3 years of college or similar higher education; 16 = college graduate or equivalent.

**Table 8:** Summary of demographic data, average over all five years. (See table notes for explanation).

	C	NC	GC	GNC	GC	NGC	NGC	NGNC
**Sex/Gender**								
Men	48.2	48.5	48.1	48.5	48.1	48.2	48.2	48.5
	(0.3)	(0.2)	(0.9)	(0.7)	(0.9)	(0.3)	(0.3)	(0.2)
Women	51.8	51.5	51.9	51.5	51.9	51.8	51.8	51.5
	(0.4)	(0.2)	(1.0)	(0.6)	(1.0)	(0.4)	(0.4)	(0.2)
**Race/Ethnicity**								
White	**52.6**	**64.5**	**65.4**	**49.2**	**65.4**	**51.6**	**51.6**	**69.7**
	**(0.3)**	**(0.3)**	**(0.9)**	**(0.7)**	**(0.9)**	**(0.3)**	**(0.3)**	**(0.2)**
Non-White	**47.4**	**35.5**	**34.6**	**50.8**	**34.6**	**48.4**	**48.4**	**30.3**
	**(0.3)**	**(0.2)**	**(1.0)**	**(0.6)**	**(1.0)**	**(0.4)**	**(0.4)**	**(0.2)**
**Age (Years)**								
18–24	**12.1**	**13.0**	**10.8**	**13.5**	**10.8**	**12.2**	**12.2**	**12.8**
	**(0.2)**	**(0.2)**	**(0.7)**	**(0.6)**	**(0.7)**	**(0.2)**	**(0.2)**	**(0.2)**
65–69	**6.3**	**6.0**	**7.6**	**5.5**	**7.6**	**6.2**	6.2	6.2
	**(0.1)**	**(0.1)**	**(0.4)**	**(0.2)**	**(0.4)**	**(0.1)**	(0.1)	(0.1)
70–74	4.7	4.6	**5.7**	**4.2**	**5.7**	**4.6**	4.6	4.7
	(0.1)	(0.0)	**(0.4)**	**(0.2)**	**(0.4)**	**(0.1)**	(0.1)	(0.0)
75–79	**3.8**	**3.4**	**4.7**	**3.0**	**4.7**	**3.8**	3.8	3.6
	**(0.1)**	**(0.1)**	**(0.4)**	**(0.2)**	**(0.4)**	**(0.1)**	(0.1)	(0.0)
80+	**4.5**	**3.9**	**5.7**	**3.1**	**5.7**	**4.4**	**4.4**	**4.1**
	**(0.1)**	**(0.0)**	**(0.3)**	**(0.1)**	**(0.3)**	**(0.2)**	**(0.2)**	**(0.1)**
**Education (Years)**								
1–8	**5.6**	**3.7**	**3.6**	**5.4**	**3.6**	**5.8**	**5.8**	**3.1**
	**(0.2)**	**(0.1)**	**(0.4)**	**(0.4)**	**(0.4)**	**(0.2)**	**(0.2)**	**(0.1)**
12	**24.9**	**26.6**	**30.0**	**25.1**	**30.0**	**24.5**	**24.5**	**27.2**
	**(0.3)**	**(0.2)**	**(0.9)**	**(0.6)**	**(0.9)**	**(0.3)**	**(0.3)**	**(0.2)**
15	**28.9**	**31.9**	32.3	31.3	**32.3**	**28.7**	**28.7**	**32.1**
	**(0.3)**	**(0.2)**	(0.9)	(0.6)	**(0.9)**	**(0.3)**	**(0.3)**	**(0.2)**
16	**31.7**	**29.1**	**24.2**	**28.5**	**24.2**	**32.3**	**32.3**	**29.3**
	**(0.3)**	**(0.2)**	**(0.7)**	**(0.6)**	**(0.7)**	**(0.3)**	**(0.3)**	**(0.2)**
**Income ($ × 10^3^)**								
<10	**6.6**	**5.3**	5.9	6.0	**5.9**	**6.7**	**6.7**	**5.0**
	**(0.2)**	**(0.1)**	(0.5)	(0.4)	**(0.5)**	**(0.2)**	**(0.2)**	**(0.1)**
<15	**5.5**	**4.6**	**6.0**	**4.8**	6.0	5.4	**5.4**	**4.5**
	**(0.2)**	**(0.1)**	**(0.5)**	**(0.3)**	(0.5)	(0.2)	**(0.2)**	**(0.1)**
<50	**12.1**	**13.6**	**14.6**	**13.3**	**14.6**	**11.9**	**11.9**	**13.7**
	**(0.2)**	**(0.2)**	**(0.7)**	**(0.5)**	**(0.7)**	**(0.2)**	**(0.2)**	**(0.2)**
<75	**13.6**	**15.4**	14.5	13.9	14.5	13.5	**13.5**	**15.9**
	**(0.2)**	**(0.2)**	(0.7)	(0.5)	(0.7)	(0.3)	**(0.3)**	**(0.2)**
>75	**36.4**	**34.6**	**27.6**	**33.0**	**27.6**	**37.1**	**37.1**	**35.2**
	**(0.3)**	**(0.2)**	**(0.9)**	**(0.6)**	**(0.9)**	**(0.3)**	**(0.3)**	**(0.2)**

Numbers represent % of respondents; numbers in bold type are significantly different at *p* < 0.05; numbers in parentheses represent 95% confidence intervals expressed as a single ± number. Comparisons are all coastal (C) vs. all non-coastal (NC); Gulf coastal GC) vs. Gulf non-coastal (GNC); Gulf coastal (GC) vs. non-Gulf coastal (NGC); and non-Gulf coastal (NGC) vs. non-Gulf, non-coastal (NGNC). Education years are: 1–8 = elementary school; 12 = high school or equivalent; 15 = 1–3 years of college or similar higher education; 16 = college graduate or equivalent. For ease of reading, comparisons for significance are made in pairs: C vs. NC, GC vs. GNC, GC vs. NGC, and NGC vs. NGNC, with alternating gray and white backgrounds to separate paired comparisons.

## Data Availability

No new data were collected in the preparation of this paper. All data used in the analyses reported here are available publicly from the U.S. Centers for Disease Control and Prevention (https://www.cdc.gov/brfss/smart/Smart_data.htm last accessed on 3 August 2021).

## Supplementary Materials

The following is available online at https://www.mdpi.com/article/10.3390/ijerph18168399/s1, Table S1: List of MMSAs included in study and years for which SMART datasets were available.

## References

[1] Gascon M., Zijlema W., Vert C., White M.P., Nieuwenhuijsen M.J. (2017). Outdoor blue spaces, human health and well-being: A systematic review of quantitative studies. Int. J. Hyg. Environ. Health.

[2] Grellier J., White M.P., Albin M., Bell S., Elliott L.R., Gascón M., Gualdi S., Mancini L., Nieuwenhuijsen M., Sarigiannis D. (2017). BlueHealth: A study programme protocol for mapping and quantifying the potential benefits to public health and well-being from Europe’s blue spaces. BMJ Open.

[3] Britton E., Kindermann G., Domegan C., Carlin C. (2018). Blue care: A systematic review of blue space interventions for health and wellbeing. Health Prom. Int..

[4] Garrett J.K., Clitherow T.J., White M.P., Wheeler B.W., Fleming L.E. (2019). Coastal proximity and mental health among urban adults in England: The moderating effect of household income. Health Place.

[5] Pasanen T.P., White M.P., Wheeler B.W., Garrett J.K., Elliott L.R. (2019). Neighbourhood blue space, health and well-being: The mediating role of different types of physical activity. Environ. Int..

[6] Elliott L.R., White M.P., Grellier J., Garrett J.K., Cirach M., Wheeler B.W., Bratman G.N., van den Bosch M.A., Ojala A., Roiko A. (2020). Research Note: Residential distance and recreational visits to coastal and inland blue spaces in eighteen countries. Landsc. Urban Plan..

[7] Hooyberg A., Roose H., Grellier J., Elliott L.R., Lonneville B., White M.P., Michels N., De Henauw S., Vandegehuchte M., Everaert G. (2020). General health and residential proximity to the coast in Belgium: Results from a cross-sectional health survey. Environ. Res..

[8] White M.P., Elliott L.R., Gascon M., Roberts B., Fleming L.E. (2020). Blue space, health and well-being: A narrative overview and synthesis of potential effects. Environ. Res..

[9] White M.P., Alcock I., Wheeler B.H., Depledge M.H. (2013). Coastal proximity, health and well-being: Results from a longitudinal panel survey. Health Place.

[10] Wheeler B.W., White M., Stahl-Timmins W., Depledge M.H. (2012). Does living by the coast improve health and wellbeing?. Health Place.

[11] Sandifer P.A., Walker A.H. (2018). Enhancing disaster resilience by reducing stress-associated health impacts. Front. Public Health.

[12] Neumann B., Vafeidis A.T., Zimmermann J., Nichols R.J. (2015). Future population growth and exposure to sea-level rise and coastal flooding—A global assessment. PLoS ONE.

[13] Sweet W., Dusek G., March D., Carbin G., Marra J. (2019). 2018 State of High Tide Flooding with a 2019 Outlook.

[14] Sinay L., Carter R.W. (2020). Climate change adaptations for coastal communities and local governments. Climate.

[15] Sandifer P.A., Scott G.I. (2021). Coastlines, coastal cities, and climate change: A perspective on urgent research needs in the United States. Front. Mar. Sci..

[16] Sandifer P., Knapp L., Lichtveld M., Manley R., Abramson D., Caffey R., Cochran D., Collier T., Ebi K., Engel L. (2021). Framework for a community health observing system for the Gulf of Mexico region: Preparing for future disasters. Front. Public Health.

[17] Sandifer P., Knapp L., Lichtveld M., Manley R., Abramson D., Caffey R., Cochran D., Collier T., Ebi K., Engel L. (2020). A Conceptual Framework for a Community Health Observing System for the Gulf of Mexico Region. Gulf of Mexico Research Initiative Core Synthesis Area 4.

[18] Fleming L.E., McDonough N., Austen M., Mee L., Moore M., Hess P., Depledge M.H., Philippart K., Bradbrook P., Smalley A. (2014). Oceans and human health: A rising tide of challenges and opportunities for Europe. Mar. Environ. Res..

[19] Scott C.H., Horton C., Brett C., Palmer E., Pipes S., Tufford D., Sandier P.A., DeLorenzo M., Pennington P.L., Porter D.E. (2019). Vibrio bacteria in aquatic ecosystems: Effects of climate change on antibiotic resistance. Encyclopedia of Water: Science, Technology, and Society.

[20] Sandifer P.A., Keener P., Scott G.I., Porter D.E., Hotaling L., Spinrad R. (2021). Oceans and human health and the new blue economy. Preparing a Workforce for the New Blue Economy.

[21] SAMHSA and Centers for Disease Control and Prevention (2013). Behavioral Health in the Gulf Coast Region Following the Deepwater Horizon Oil Spill.

[22] Fan A.Z., Prescott M.R., Zhao G., Gotway C.A., Galea S. (2014). Individual and community-level determinants of mental and physical health after the Deepwater Horizon oil spill: Findings from the Gulf States Population Survey. J. Behav. Health Serv. Res..

[23] Gould D.W., Teich J.L., Pemberton M.R., Pierannunzi C., Larson S. (2014). Behavioral health in the Gulf coast following the Deepwater Horizon oil spill: Findings from two Federal surveys. J. Behav. Health Serv. Res..

[24] LaFlamme D.M., van der Slice J.A. (2014). Using the Behavioral Risk Factor Sureveillance System (BRFSS) for exposure tracking: Experiences from Washington State. Environ. Health Perspect..

[25] Kroenke (2009). K.; Strine, T.W.; Spitzer, R.L.; Williams, J.B.W.; Berry, J.T.; Mokdad, A.H. The PHQ-8 as a measure of current depression in the general population. J. Affect. Dis..

[26] Khalil G.M., Crawford C.A.G. (2015). A bibliometric analysis of U.S.-based research on the Behavioral Risk Factor Surveillance System. Am. J. Prev. Med..

[27] Tomitaka S., Kawasaki Y., Ide K., Akutagawa M., Yamada H., Yutaka O., Furukawa T.A. (2017). Item response patterns on the patient health questionnaire-8 in a nationally representative sample of US adults. Front. Psychiatry.

[28] Bor J., Venkataramani A.S., Williams D.K., Tsai A.C. (2018). Police killings and their spillover effects on the mental health of black Americans: A population-based, quasi-experimental study. Lancet.

[29] Zahran H.S., Koban R., Moriarty D.G., Zack M.M., Holt J., Donehoo R. (2005). Health-related quality of life surveillance—United States, 1993–2002. Morb. Mortal. Wkly. Rep..

[30] Slabaugh S.L., Shah M., Zack N., Happe L., Cordier T., Havens E., Davidson E., Miao M., Prewitt T., Jia H. (2017). Leveraging health-related quality of life in population health management: The case for healthy days. Popul. Health Manag..

[31] CDC (Centers for Disease Control and Prevention) (2000). Measuring Healthy Days.

[32] IOM (Institute of Medicine) (2009). State of the USA Health Indicators: Letter Report.

[33] Shockey T.M., Zack M., Sussell A. (2017). Health-related quality of life among US workers: Variability across occupation groups. Am. J. Public Health.

[34] Chimed-Ochir O., Mine K., Fujino Y. (2019). Pain, unhealthy days and poor perceived health among Japanese workers. J. Occup. Health.

[35] (2016). US Census. https://www.census.gov/library/visualizations/2016/comm/acs-rural-urban.html.

[36] Asada Y., Whipp A., Kindig D., Billard B., Rudolph B. (2014). Inequalities in multiple health outcomes by education, sex, and race in 93 US counties: Why we should measure them all. Int. J. Equity Health..

[37] Cordier T., Song Y., Cambon J., Haugh G.S., Steffen M., Hardy P., Staehly M., Hagan A., Gopal V., Tye P.D. (2018). A bold goal: More healthy days through improved community health. Popul. Health Manag..

[38] Fry C.E., Nikpay S.S., Leslie E., Buntin M.B. (2018). Evaluating community-based health improvement programs. Health Aff..

[39] Ha H. (2018). Using geographically weighted regression for social inequality analysis: Association between mentally unhealthy days (MUDs) and socioeconomic status (SES) in U.S. counties. Int. J. Environ. Health Res..

[40] Ha H., Shao W. (2019). A spatial epidemiology case study of mentally unhealthy days (MUDs): Air pollution, community resilience, and sunlight perspectives. Int. J. Environ. Health Res..

[41] Caraballo R.S., Rice K.L., Neff L.J., Garrett B.E. (2019). Social and physical characteristics associated with adult current cigarette smoking. Prev. Chronic Dis..

[42] NOAA (National Oceanic and Atmospheric Administration) (2017). Coastal County Definitions. https://coast.noaa.gov/data/digitalcoast/pdf/qrt-coastal-county-definitions.pdf.

[43] Pickard B.R., Daniel J., Mehaffey M., Jackson L.E., Neale A. (2015). EnviroAtlas: A new geospatial tool to foster ecosystem services science and resource management. Ecosyst. Serv..

[44] USEPA (2016). Dasymetric Allocation of Population, Raster Dataset.

[45] Wessel P., Smith W.H.F. (1996). A global, self-consistent, hierarchical, high-resolution shoreline database. J. Geophysical Res.: Solid Earth..

[46] Cree R.A., Oloro C.A., Zack M.M., Carbone E. (2020). Frequent mental distress among adults by disability status, disability type, and selected characteristics—United States, 2018. MMWR.

[47] Zhao G., Okoro C.A., Hsia J., Town M. (2018). Self-perceived poor/fair health, frequent mental distress, and health insurance status among working-aged US adults. Prev. Chronic Dis..

[48] Strine T.W., Chapman D.P., Kobau R., Balluz L., Mokdad A. (2004). Depression, anxiety, and physical impairments and quality of life in the U.S. noninstitutionalized population. Psychiatr. Serv..

[49] Zhou H., Siegel P.Z., Barile J., Njai R.S., Thompson W.W., Kent C., Liao Y. (2016). Models for count data with an application to Healthy Days measures: Are you driving in screws with a hammer?. Prev. Chronic Dis..

[50] Brereton F., Clinch J.P., Ferreira S. (2008). Happiness, geography and the environment. Ecol. Econ..

[51] Nutsford D., Pearson A.L., Kingham S., Reitsma F. (2016). Residential exposure to visible blue space (but not green space) associated with lower psychological distress in a capital city. Health Place.

[52] Dempsey S., Devine M.T., Gillespie T., Lyons S., Nolan A. (2018). Coastal blue space and depression in older adults. Health Place.

[53] Coleman T., Kearns R. (2015). The role of bluespaces in experiencing place, aging and well-being: Insights from Waiheke Island, New Zealand. Health Place.

[54] Garrett J., White M.P., Huang J., Ng S., Hug Z., Leung C., Tse S., Fung F., Elliott L.R., Depledge M.H. (2019). The association between blue space, health and well-being in Hong Kong. Health Place.

[55] Lichtveld M.Y., Arosemena F.A. (2014). Resilience in the aftermath of the Gulf of Mexico oil spill: An academic-community partnership to improve health education, social support, access to care, and disaster preparedness. Int. Oil Spill Conf. Proc..

[56] Anderson D.M., Fensin E., Gobler C.J., Hoeglund A.E., Hubbard K.A., Kulis D.M., Landsberg J.H., Lefebvre K.A., Provoost P., Richlen M.L. (2021). Marine harmful algal blooms in the United States: History current status and future trends. Harmful Algae.

[57] Mishra H.S., Bell S., Vassiljev P., Kuhlmann F., Niin G., Grellier J. (2020). The development of a tool for assessing the environmental qualities of urban blue spaces. Urban Forest. Urban Green..

[58] Annerstedt M., Ostergren P.O., Bjork J., Grahn P., Skarback E., Wahrborg P. (2012). Green qualities in the neighbourhood and mental health—results from a longitudinal cohort study in Southern Sweden. BMC Public Health.

[59] Oates G.R., Jackson B.E., Partridge E.E., Singh K.P., Fouad M.N., Bae S. (2017). Sociodemographic patterns of chronic disease: How the mid-South region compares to the rest of the country. Am. J. Prev. Med..

[60] Parker A.M., Edelman A.F., Carman K.G., Finucane M.L. (2019). On the need for prospective disaster survey panels. Dis. Med. Public Health Prep..

