# Supplementary material for: Is Living in a U.S. Coastal City Good for One’s Health?
Source: Int J Environ Res Public Health. 2021 Aug 9;18(16):8399. doi: 10.3390/ijerph18168399 (PMC8393764; doi:10.3390/ijerph18168399)
Supplement: Supplementary file 1 [file ijerph-18-08399-s001.zip › ijerph-1295325-supplementary.pdf]

Supplemental Table S1: List of MMSAs included in study and years for which SMART datasets were available.

Gulf of Mexico Coastal MMSAs

Corpus Christi, TX, Metropolitan Statistical Area: 2014-2017
Crestview-Fort Walton Beach-Destin, FL, Metropolitan Statistical Area: 2013, 2016, 2017
Gulfport-Biloxi-Pascagoula, MS, Metropolitan Statistical Area: 2013, 2015
New Orleans-Metairie, LA, Metropolitan Statistical Area: all years
North Port-Sarasota-Bradenton, FL, Metropolitan Statistical Area: 2013, 2014, 2016, 2017
Panama City, FL, Metropolitan Statistical Area: 2013, 2016, 2017
Pensacola-Ferry Pass-Brent, FL, Metropolitan Statistical Area: 2013, 2016, 2017
Tampa-St. Petersburg-Clearwater, FL, Metropolitan Statistical Area: all years

Non-Gulf of Mexico Coastal MMSAs

Baltimore-Columbia-Towson, MD, Metropolitan Statistical Area: all years
Boston, MA, Metropolitan Division: all years
Buffalo-Cheektowaga-Niagara Falls, NY, Metropolitan Statistical Area: 2013, 2015-2017
Cambridge-Newton-Framingham, MA, Metropolitan Division: all years
Charleston-North Charleston, SC, Metropolitan Statistical Area: all years
Chicago-Naperville-Elgin, IL-IN-WI, Metropolitan Statistical Area: all years
Cleveland-Elyria, OH, Metropolitan Statistical Area: all years
Deltona-Daytona Beach-Ormond Beach, FL, Metropolitan Statistical Area: 2013, 2016, 2017
Duluth, MN-WI, Metropolitan Statistical Area: all years
Hilton Head Island-Bluffton-Beaufort, SC, Metropolitan Statistical Area: all years
Los Angeles-Long Beach-Anaheim, CA, Metropolitan Statistical Area: all years
Miami-Fort Lauderdale-West Palm Beach, FL, Metropolitan Statistical Area: all years
Milwaukee-Waukesha-West Allis, WI, Metropolitan Statistical Area: all years
Myrtle Beach-Conway-North Myrtle Beach, SC-NC, Metropolitan Statistical Area: all years
Nassau County-Suffolk County, NY, Metropolitan Division: all years
New York-Jersey City-White Plains, NY-NJ, Metropolitan Division: all years
Newark, NJ-PA, Metropolitan Division: all years
Oakland-Hayward-Berkeley, CA, Metropolitan Division: all years
Port St. Lucie, FL, Metropolitan Statistical Area: 2013, 2016, 2017
Portland-South Portland, ME, Metropolitan Statistical Area: all years
Providence-Warwick, RI-MA, Metropolitan Statistical Area: all years
Rochester, NY, Metropolitan Statistical Area: 2013, 2015-2017
Salisbury, MD-DE, Metropolitan Statistical Area: all years
San Francisco-Redwood City-South San Francisco, CA, Metropolitan Division: 2013, 2015, 2016
San Jose-Sunnyvale-Santa Clara, CA, Metropolitan Statistical Area: 2015, 2016
Seattle-Bellevue-Everett, WA, Metropolitan Division: all years
Toledo, OH, Metropolitan Statistical Area: all years
Virginia Beach-Norfolk-Newport News, VA-NC, Metropolitan Statistical Area: all years
Wilmington, DE-MD-NJ, Metropolitan Division: all years

1.

Gulf of Mexico Non-Coastal MMSAs

Atlanta-Sandy Springs-Roswell, GA, Metropolitan Statistical Area: all years
Augusta-Richmond County, GA-SC, Metropolitan Statistical Area: all years
Austin-Round Rock, TX, Metropolitan Statistical Area: all years: all years
Baton Rouge, LA, Metropolitan Statistical Area: all years
Birmingham-Hoover, AL, Metropolitan Statistical Area: all years
College Station-Bryan, TX, Metropolitan Statistical Area: 2014-2017
Dallas-Plano-Irving, TX, Metropolitan Division: all years
Fort Worth-Arlington, TX, Metropolitan Division: all years
Gainesville, FL, Metropolitan Statistical Area: 2013, 2016, 2017
Houston-The Woodlands-Sugar Land, TX, Metropolitan Statistical Area: all years
Jackson, MS, Metropolitan Statistical Area: 2013, 2015-2017
Lafayette, LA, Metropolitan Statistical Area: 2014
Montgomery, AL, Metropolitan Statistical Area: 2014
Orlando-Kissimmee-Sanford, FL, Metropolitan Statistical Area: all years
San Antonio-New Braunfels, TX, Metropolitan Statistical Area: all years
Shreveport-Bossier City, LA, Metropolitan Statistical Area: 2013, 2014
Tallahassee, FL, Metropolitan Statistical Area: 2013, 2016, 2017
Tuscaloosa, AL, Metropolitan Statistical Area: 2014-2017

Non-Gulf of Mexico Non-Coastal MMSAs

Aberdeen, SD, Micropolitan Statistical Area: 2014, 2015, 2017
Akron, OH, Metropolitan Statistical Area: 2013, 2015
Albany-Schenectady-Troy, NY, Metropolitan Statistical Area: 2015-2017
Albuquerque, NM, Metropolitan Statistical Area: all years
Allentown-Bethlehem-Easton, PA-NJ, Metropolitan Statistical Area: all years
Beckley, WV, Metropolitan Statistical Area: 2016
Berlin, NH-VT, Micropolitan Statistical Area: 2014, 2016
Billings, MT, Metropolitan Statistical Area: all years
Binghamton, NY, Metropolitan Statistical Area: 2016
Bismarck, ND, Metropolitan Statistical Area: all years
Boise City, ID, Metropolitan Statistical Area: all years
Burlington-South Burlington, VT, Metropolitan Statistical Area: all years
Camden, NJ, Metropolitan Division: all years
Cedar Rapids, IA, Metropolitan Statistical Area: 2013, 2014, 2016, 2017
Charleston, WV, Metropolitan Statistical Area: all years
Charlotte-Concord-Gastonia, NC-SC, Metropolitan Statistical Area: all years
Chattanooga, TN-GA, Metropolitan Statistical Area: 2013, 2016
Cincinnati, OH-KY-IN, Metropolitan Statistical Area: all years
Claremont-Lebanon, NH-VT, Micropolitan Statistical Area: all years
Colorado Springs, CO, Metropolitan Statistical Area: all years

Columbia, SC, Metropolitan Statistical Area: all years
Columbus, OH, Metropolitan Statistical Area: all years
Cumberland, MD-WV, Metropolitan Statistical Area: 2016
Davenport-Moline-Rock Island, IA-IL, Metropolitan Statistical Area: 2013
Dayton, OH, Metropolitan Statistical Area: all years
Denver-Aurora-Lakewood, CO, Metropolitan Statistical Area: all years
Des Moines-West Des Moines, IA, Metropolitan Statistical Area: all years
Durham-Chapel Hill, NC, Metropolitan Statistical Area: 2013
Dutchess County-Putnam County, NY, Metropolitan Division: 2016
El Paso, TX, Metropolitan Statistical Area: all years
Evansville, IN-KY, Metropolitan Statistical Area: 2013, 2014, 2017
Fargo, ND-MN, Metropolitan Statistical Area: all years
Fayetteville-Springdale-Rogers, AR-MO, Metropolitan Statistical Area: all years
Florence, SC, Metropolitan Statistical Area: 2015, 2017
Fort Smith, AR-OK, Metropolitan Statistical Area: 2013
Fort Wayne, IN, Metropolitan Statistical Area: 2013, 2014, 2016, 2017
Glens Falls, NY, Metropolitan Statistical Area: 2016
Grand Forks, ND-MN, Metropolitan Statistical Area: 2013, 2016, 2017
Grand Island, NE, Metropolitan Statistical Area: all years
Grand Rapids-Wyoming, MI, Metropolitan Statistical Area: all years
Greensboro-High Point, NC, Metropolitan Statistical Area: 2013, 2014
Greenville-Anderson-Mauldin, SC, Metropolitan Statistical Area: all years
Hagerstown-Martinsburg, MD-WV, Metropolitan Statistical Area: all years
Hartford-West Hartford-East Hartford, CT, Metropolitan Statistical Area: all years
Huntington-Ashland, WV-KY-OH, Metropolitan Statistical Area: all years
Idaho Falls, ID, Metropolitan Statistical Area: 2013-2015
Indianapolis-Carmel-Anderson, IN, Metropolitan Statistical Area: all years
Jacksonville, FL, Metropolitan Statistical Area: all years
Kansas City, MO-KS, Metropolitan Statistical Area: all years
Kennewick-Richland, WA, Metropolitan Statistical Area: 2015
Kingsport-Bristol-Bristol, TN-VA, Metropolitan Statistical Area: all years
Knoxville, TN, Metropolitan Statistical Area: all years
Lansing-East Lansing, MI, Metropolitan Statistical Area: 2013, 2016, 2017
Lexington-Fayette, KY, Metropolitan Statistical Area: 2013, 2014, 2017
Lincoln, NE, Metropolitan Statistical Area: all years
Little Rock-North Little Rock-Conway, AR, Metropolitan Statistical Area: all years
Logan, UT-ID, Metropolitan Statistical Area: 2013-2016
Louisville/Jefferson County, KY-IN, Metropolitan Statistical Area: all years
Lubbock, TX, Metropolitan Statistical Area: 2013
Madison, WI, Metropolitan Statistical Area: 2014
Manhattan, KS, Metropolitan Statistical Area: 2013, 2015, 2017

Memphis, TN-MS-AR, Metropolitan Statistical Area: all years
Minneapolis-St. Paul-Bloomington, MN-WI, Metropolitan Statistical Area: all years
Minot, ND, Micropolitan Statistical Area: all years
Montgomery County-Bucks County-Chester County, PA, Metropolitan Division: all years
Nashville-Davidson--Murfreesboro--Franklin, TN, Metropolitan Statistical Area: all years
Norfolk, NE, Micropolitan Statistical Area: 2013-2016
North Platte, NE, Micropolitan Statistical Area: all years
Ogden-Clearfield, UT, Metropolitan Statistical Area: all years
Oklahoma City, OK, Metropolitan Statistical Area: all years
Omaha-Council Bluffs, NE-IA, Metropolitan Statistical Area: all years
Philadelphia, PA, Metropolitan Division: all years
Phoenix-Mesa-Scottsdale, AZ, Metropolitan Statistical Area: all years
Pittsburgh, PA, Metropolitan Statistical Area: all years
Portland-Vancouver-Hillsboro, OR-WA, Metropolitan Statistical Area: all years
Provo-Orem, UT, Metropolitan Statistical Area: all years
Raleigh, NC, Metropolitan Statistical Area: 2013-2016
Rapid City, SD, Metropolitan Statistical Area: all years
Reno, NV, Metropolitan Statistical Area: all years
Richmond, VA, Metropolitan Statistical Area: all years
Riverside-San Bernardino-Ontario, CA, Metropolitan Statistical Area: all years
Roanoke, VA, Metropolitan Statistical Area: 2014
Rochester, MN, Metropolitan Statistical Area: 2014-2017
Rockingham County-Strafford County, NH, Metropolitan Division: all years
Sacramento--Roseville--Arden-Arcade, CA, Metropolitan Statistical Area: all years
Salem, OR, Metropolitan Statistical Area: 2013, 2016
Salina, KS, Micropolitan Statistical Area: 2013, 2015, 2017
Salt Lake City, UT, Metropolitan Statistical Area: all years
Scottsbluff, NE, Micropolitan Statistical Area: all years
Scranton--Wilkes-Barre--Hazleton, PA, Metropolitan Statistical Area: 2013
Silver Spring-Frederick-Rockville, MD, Metropolitan Division: all years
Sioux City, IA-NE-SD, Metropolitan Statistical Area: all years
Sioux Falls, SD, Metropolitan Statistical Area: all years
South Bend-Mishawaka, IN-MI, Metropolitan Statistical Area: 2017
Spartanburg, SC, Metropolitan Statistical Area: all years
Spokane-Spokane Valley, WA, Metropolitan Statistical Area: all years
Springfield, MA, Metropolitan Statistical Area: all years
St. Cloud, MN, Metropolitan Statistical Area: 2014-2017
St. Louis, MO-IL, Metropolitan Statistical Area: all years
Syracuse, NY, Metropolitan Statistical Area: 2016
Topeka, KS, Metropolitan Statistical Area: all years
Tulsa, OK, Metropolitan Statistical Area: all years

Utica-Rome, NY, Metropolitan Statistical Area: 2016
Warren-Troy-Farmington Hills, MI, Metropolitan Division: all years
Washington-Arlington-Alexandria, DC-VA-MD-WV, Metropolitan Division: all years
Wichita Falls, TX, Metropolitan Statistical Area: 2014-2017
Wichita, KS, Metropolitan Statistical Area: all years
Winston-Salem, NC, Metropolitan Statistical Area: 2013
Worcester, MA-CT, Metropolitan Statistical Area: all years
Youngstown-Warren-Boardman, OH-PA, Metropolitan Statistical Area: 2014
